# MAGEH1 interacts with GADD45G and induces renal tubular cell apoptosis

**DOI:** 10.1371/journal.pone.0260135

**Published:** 2021-11-17

**Authors:** Gyu-Tae Shin, Ji Eun Park, Min-Jeong Lee

**Affiliations:** Department of Nephrology, Ajou University School of Medicine, Suwon, Korea; Wake Forest School of Medicine: Wake Forest University School of Medicine, UNITED STATES

## Abstract

**Background:**

Melanoma-associated antigen H1 (MAGEH1) is a protein that belongs to melanoma-associated antigen (MAGE) superfamily. Growth arrest and DNA damage 45G (GADD45G) is a member of the DNA damage-inducible gene family which responds to environmental stresses. We have previously shown that GADD45G is a protein that promotes apoptosis of renal tubular cells in response to a nephrotoxic injury. In this study, we show evidence that MAGEH1 interacts with GADD45G and is involved in the induction of nephrotoxin-induced apoptosis of renal tubular cells.

**Methods:**

Primary human renal tubular epithelial (HRE) cells and human kidney 2 **(**HK-2) cells were used in this study. To produce stable cell lines in which MAGEH1 expression was silenced, HRE cells were transduced with a lentiviral vector encoding a single guide RNA construct targeting the MAGEH1 gene. To knockdown GADD45G expression in HRE cells, a vector containing short hairpin RNA (shRNA) was used. We used short interfering RNAs (siRNA) to achieve transient silencing of genes in HK-2 cells. Recombinant adenoviruses were synthesized to overexpress MAGEH1 and GADD45G proteins. Human protein microarray was used to identify proteins that binds to GADD45G. Co-immunoprecipitation assays were then performed to confirm microarray results. Cell death was induced by cyclosporine A (CsA). Real-time quantitative PCR assay was used to evaluate gene expression levels. The degree of apoptosis and necrosis of cultured cells was evaluated by flow cytometry. Expression levels of caspases were examined using western blot analysis.

**Results:**

We found that GADD45G bound to one protein spotted in the protein microarray, which was subsequently identified as MAGEH1. We confirmed the interaction between GADD45G and MAGEH1 protein using the co-immunoprecipitation assay. *MAGEH1* gene expression was not altered by CsA-induced cytotoxic injury, whereas *GADD45G* gene expression was increased significantly upon CsA treatment. *MAGEH1* expression was significantly downregulated in GADD45G knockdown HRE stable cells suggesting that MAGEH1 expression may be dependent on GADD45G expression. CsA-induced apoptosis was significantly reduced in MAGEH1 knockdown HRE stable cells which led to an increased survival of these cells. Similar results were observed in GADD45G knockdown HRE stable cells. Accordingly, CsA-induced apoptosis was significantly decreased in MAGEH1 siRNA and GADD45G siRNA transfected HK-2 cells. CsA-induced activation of caspase-7 and caspase-9 was inhibited in MAGEH1 knockdown HRE stable cells, and similarly in GADD45G knockdown HRE stable cells.

**Conclusions:**

To the best of our knowledge, this is the first study to show that MAGEH1 interacts with GADD45G and that MAGEH1 is involved in caspase-dependent apoptosis of renal tubular cells induced by nephrotoxic drugs.

## Introduction

Growth arrest and DNA damage 45G (GADD45G) is a family of proteins involved in DNA damage response and cell growth arrest [1,2]. We have previously shown that GADD45G promotes apoptosis leading to acute and chronic kidney injuries [3–5].

Melanoma-associated antigen H1 (MAGEH1) is a protein that belongs to melanoma-associated antigen (MAGE) superfamily. The first member of the human MAGE family, MAGE-1, was identified from a human melanoma cell line, which was later renamed MAGEA1 upon the discovery of additional gene family members [6–9]. The MAGE gene family has been categorized into two types (type I and type II) based on tissue expression pattern. Type I MAGEs include MAGE-A, -B, and -C subfamily members. They are expressed in various tumor cells but completely silent in normal tissues except male germ cells. Because of such exclusive expression on tumor cells, type I MAGEs have attracted a particular attention as biomarkers in cancer and potential targets of immunotherapies [10]. In contrast, type II MAGEs, which MAGEH1 belongs to, are expressed throughout a variety of normal tissues in the body. So far, about a dozen members of type II MAGEs have been identified including MAGE-D, -E, -F, -G, -H, -I, -J, -K, -L and necdin [11]. Although roles of MAGE proteins in cell activities are largely unknown, studies are beginning to provide insights into MAGE functions, revealing that MAGE proteins regulate diverse cellular and developmental pathways [12]. Accordingly, recent evidence has indicated that MAGE proteins may play a role in renal pathophysiology: It has been reported that MAGED2 is essential for fetal renal salt reabsorption and that MAGED2 mutations cause transient form of antenatal Bartter’s syndrome [13]. Other investigators have reported that five MAGE genes (MAGED1, MAGED2, MAGED3, MAGEH1, MAGEE1) are expressed in healthy adult mouse kidneys and that MAGED2 is upregulated during an experimental acute kidney injury [14].

In our efforts to elucidate some mechanistic details of GADD45G-induced apoptosis, we found that GADD45G binds to MAGEH1, prompting us to investigate the role of MAGEH1 in renal tubular cell death. Here, we present novel findings showing that MAGEH1 interacts with GADD45G and is involved in the induction of apoptosis of renal tubular cells.

## Materials and methods

### Identification of GADD45G binding proteins

To identify proteins that binds to GADD45G, we used HuProt human protein microarray (CDI Labs, Mayaguez, Puerto Rico) containing over 19,000 full-length recombinant human proteins. The microarray was incubated with blocking buffer (5% BSA in PBS with 0.05% tween 20) for 2 hours and then 3 μg of biotinylated anti-GADD45G antibody (Santa Cruz Biotechnology, Dallas, TX) was added onto the array. Subsequently, 1 μg of streptavidin-fluorescence (Alexa-Fluor 635 nm, red) was added to obtain antibody cross−reactivity data. After washing, the microarray was treated with 3 μg of GADD45G protein (Abcam, Cambridge, MA) for 12 hours at 4°C and then incubated with an anti-GADD45G antibody for 2 hours at 4°C. The array was then incubated with 1 μg of streptavidin-fluorescence (Alexa-Fluor 532 nm, green). Microarray results were obtained by scanning using a GenePix4100A microarray laser scanner (Molecular Devices, Sunnyvale, CA).

### Cell culture

Primary human renal tubular epithelial (HRE) cells were purchased from Lonza (Walkersville, MD) and maintained in Renal Epithelial Basal Medium supplemented with 10% FBS and SingleQuots kits (Lonza). Human kidney 2 (HK-2) cells, a proximal tubular cell line derived from normal kidney, were purchased from American Type Culture Collection (ATCC) (Manassas, VA) and maintained in DMEM/F12 medium supplemented with 10% FBS (Thermo Fisher Scientific, Waltham, MA). Cell cultures were tested for the presence of Mycoplasma using a Mycoplasma detection kit (MycoStrip) (InvivoGen, San Diego CA).

### Construction of recombinant adenoviruses

The shuttle vector for making recombinant adenoviruses containing the *GADD45G* gene (Ad-GADD45G) was synthesized based on Gateway technology using ViraPower Adenoviral Gateway Expression Kit (Invitrogen) as per the manufacturer’s instructions. The human GADD45G full-length open reading frame (ORF) clone (Ultimate ORF clone, Invitrogen) was provided in an entry vector (Gateway pENTR221, Invitrogen). The GADD45G ORF was cloned into the destination vector pAd/CMV/V5-DEST by using LR Clonase II enzyme (Invitrogen) to synthesize pAd/CMV/V5-GADD45G. pAd/CMV/V5-GADD45G was then digested with Pac I enzyme and transfected into HEK293A cells using Lipofectamine 2000 (Invitrogen) to produce recombinant adenoviruses (Ad-GADD45G). For use as controls, pAd/CMV/V5/lacZ for β-galactosidase expression was provided with the kit and recombinant adenoviruses containing the *lacZ* gene (Ad-LacZ) were generated. The shuttle vector for making recombinant adenoviruses expressing hemagglutinin (HA)-tagged MAGEH1 proteins (Ad-HA-MAGEH1) was synthesized by Applied Biological Materials (Ferndale, WA) For purification and concentration, the Adeno-X maxi purification kit (Takara, Mountain View, CA) was used. For titration, HEK293A cells infected with recombinant adenoviruses were detected using an antibody specific for the adenovirus hexon protein with the Adeno-X rapid titer kit (Takara).

### Protein extraction

Cultured cell monolayers were scraped in lysis solution (Cell signaling, Danvers, MA) containing protease inhibitors (Protease Inhibitor Cocktail) (Roche Life Science, Indianapolis, IN) and phosphatase inhibitors (PhosStop) (Roche Life Science). Homogenates were incubated on ice for 30 minutes, and then centrifuged to pellet cell debris at 13,000 rpm for 10 minutes at 4°C. Protein was quantified using the Bradford dye-binding method (Bio-Rad, Hercules, CA).

### Co-immunoprecipitation

HRE cells were infected with Ad-GADD45G and Ad-HA-MAGEH1 simultaneously at a multiplicity of infection (MOI) of 250. Proteins were then extracted 48 hours later. Five-hundred microgram of proteins were precleared using 50 μl Protein G agarose (Sigma−Aldrich), and then the supernatant was incubated with 2 μg primary antibody for GADD45G (mouse monoclonal, Santa Cruz Biotechnology) and 50 μl Protein G agarose overnight. Immunoprecipitated proteins were eluted using SDS-sample buffer, separated by SDS-PAGE, and blotted with an anti-MAGEH1 antibody (rabbit polyclonal) (Thermo Fisher Scientific, Rockford, IL), followed by incubation with anti-rabbit secondary antibodies. Non-immune mouse IgG antibodies (Santa Cruz Biotechnology) were used as a negative control. In a reciprocal fashion, proteins were immunoprecipitated with an anti-MAGEH1 antibody and then immunoblotted using the anti-GADD45G antibody.

### Immunofluorescence staining

HRE cells plated on Lab-Tek Chamber slides (Thermo Fisher Scientific) were fixed with 4% paraformaldehyde, and permeabilized with 0.1% Triton X-100 in PBS at room temperature for 15 min. Cells were blocked with 1% BSA in PBS at room temperature for 30 minutes, and then incubated with primary antibodies directed against the HA epitope (MAGEH1) (rabbit polyclonal) (1:800 dilution; Thermo Fisher Scientific) and GADD45G (mouse monoclonal) (1:50 dilution; Santacruz) at 4°C overnight. Signals were detected with Alexa Fluor 488 (1:200 dilution; Thermo Fisher Scientific) and Alexa Fluor 594 (1:200 dilution; Thermo Fisher Scientific), respectively. Then, cells were counterstained with 4′,6-Diamidino-2-phenylindole dihydrochloride (DAPI) (Thermo Fisher Scientific), and visualized with laser confocal microscopy (Nicon A1R HD25).

### Quantitative real-time polymerase chain reaction (qPCR)

Samples were homogenized in Trizol reagents (Invitrogen). RNA was extracted with chloroform, precipitated with isopropanol, washed with 75% ethanol, and then re-dissolved in TE buffer. The isolated RNA was quantified spectrophotometrically. RNA integrity was assessed by an Agilent 2100 Bioanalyzer using the Agilent RNA 6000 Nano Kit (Agilent, Santa Clara, CA). RNA Integrity Number (RIN) of 8 or above was considered acceptable. After removing contaminating DNA from the isolated RNA using DNase I (Invitrogen), 2 μg of total RNA was reverse transcribed into cDNA in the reaction mixtures containing Moloney murine leukemia virus reverse transcriptase and random hexanucleotide primers. To calculate amplification efficiency, cDNA was prepared from GADD45G or MAGEH1 overexpressing HRE cells and 10-fold dilution series were made. C_T_ values were plotted against the logarithm of cDNA dilutions to construct standard curves, and PCR efficiency (E) was calculated using the equation: E = -1+10^(-1/standard curve slope)^. PCR efficiency of all primer sets was considered acceptable: MAGEH1 (slope, -3.38; E, 97.3%); GADD45G (slope, -3.36; E, 98.19%); GAPDH (slope, -3.35; E, 98.84%). The amplification reactions were performed in a total volume of 25 μl, containing 2 μl of template DNA, 12.5 μl of TB Green Premix Ex Taq II PCR Master Mix (Takara), 1.25 μl each of (0.5–0.75 μM) forward and reverse primer, and 8 μl of sterile water using a 7500 Real Time PCR System (Applied Biosystems) using the following parameters: an initial denaturation at 95°C for 30 seconds, followed by PCR at 95° C for 5 seconds and 60°C for 30 seconds for 40 cycles. All expression levels were normalized to those of glyceraldehyde 3-phosphate dehydrogenase (GAPDH). The 2^-△△*C*^_T_ method was used to analyze the relative changes in gene expression from qPCR experiments [15]. Primers used in this study are shown in Table 1.

**Table 1:** Primers used in qPCR.

Primers expected size	Sequences	GenBank Accession
MAGEH1 144bp	F: 5′-GTGCTGGGGAAGTTAGGAATG-3′ R: 5′-GAACTCATACTCCACCGGACT-3′	NM_014061
GADD45G 116bp	F: 5′-ACACAGTTCCGGAAAGCACA-3′ R: 5′-TTTGGCTGACTCGTAGACGC-3′	NM_006705
GAPDH 109bp	F: 5′-TATAAATTGAGCCCGCAGCC-3′ R: 5′-CCATGGTGTCTGAGCGATGT-3′	NM_002046

F, forward primer; R, reverse primer; MAGEH1, melanoma-associated antigen H1; GADD45G, growth arrest and DNA damage 45G; GAPDH, glyceraldehyde-3-phosphate dehydrogenase.

### Construction of MAGEH1 knockdown HRE stable cell lines

To knockdown MAGEH1 expression in HRE cells, we used a lentiviral vector containing single guide RNAs (sgRNA) composed of target sequence CTGCGGCTCTCGCATTACGG, Streptococcus pyogenes CRISPR-associated protein 9 (SpCas9) nuclease gene, and puromycin resistance gene (Applied Biological Materials, Ferndale, WA). The negative control lentiviral vector contained scrambled sgRNA which had no homology to known gene sequences (Applied Biological Materials). HRE cells were infected with each lentivirus in the presence of 4 μg/ml polybrene and then the cells were selected using 3 μg/ml puromycin (Invivogen, San Diego, CA) to generate stable cell lines expressing sgRNA constructs targeting the *MAGEH1* gene (sgMAGEH HRE cells), or no known genes (sgCon HRE cells).

### Construction of GADD45G knockdown HRE stable cell lines

To knockdown GADD45G expression in HRE cells, we used a vector containing short hairpin RNA (shRNA) composed of target sequence CGTCTACGAGTCAGCCAAAGT, loop CTTCCTGTCA, U1 promoter, and puromycin resistance gene (SA bioscience, Frederick MD). The negative control vector contained the insert sequence GGAATCTCATTCGATGCATAC which had no homology to known gene sequences. HRE cells were transfected with each vector using SureFECT transfection reagent (SA bioscience) and the cells were selected using 3 μg/ml puromycin (Invivogen, San Diego, CA) to generate stable cell lines expressing shRNA constructs targeting the *GADD45G* gene (shGADD HRE cells), or no known genes (shCon HRE cells).

### Transient RNA interference in HK-2 cells

We used short interfering RNAs (siRNA) to silence *MAGEH1* and *GADD45G* gene expression in HK-2 cells. GADD45G, MAGEH1 and negative control siRNA reagents were purchased from Bioneer (Daejon, Korea) (Table 2). HK-2 cells were plated to become 60–80% confluent at the time of siRNA transfection, and then siRNA was introduced into HK-2 cells using Lipofectamine RNAiMAX (Invitrogen) following the manufacturer’s instructions.

**Table 2:** siRNAs for silencing of gene expression in HK-2 cells.

siRNA	Target sequence	GenBank Accession
MAGEH1	5′-CAGTGATCATTGTTCAACT-3′	NM_014061
GADD45G	5′-CGCTTGTGGATAACTAGCT-3′	NM_006705

MAGEH1, Melanoma-associated antigen H1; GADD45G, Growth arrest and DNA damage 45G.

### Detection of apoptosis and necrosis by flow cytometry

To induce cell death, HRE and HK-2 cells were treated with cyclosporine A (CsA) (Sigma-Aldrich, St. Louis, MO) for 24 to 48 hours. CsA was dissolved in ethanol to yield a 10 mg/ml stock and used at the concentrations of 12.5 to 25 μg/ml. Cells were harvested by trypsinization, pooled with the culture medium containing floating cells, collected by centrifugation, incubated with Annexin V-FITC conjugate and propidium iodide (PI) for 10 minutes using an apoptosis detection kit (APOAF) (Sigma-Aldrich), and then analyzed by flow cytometry. Percentages of apoptosis and necrosis were evaluated by plotting cell staining by Annexin V (x-axis) vs. PI (y-axis). The upper left quadrant (PI positive and Annexin V negative, Q1) represents necrotic/non-viable cells. The upper right quadrant (PI positive and Annexin V positive, Q2) represents necrotic/late apoptotic cells. The lower left quadrant (PI and Annexin V negative, Q3) represents live cells and the lower right quadrant (PI negative and Annexin V positive, Q4) represents early apoptotic cells.

### Western blot analysis for caspases

Equal amounts of total protein (10–20 μg) were subjected to SDS-PAGE using 10 to 15% acrylamide gels, transferred to a polyvinylidene difluoride (PVDF) membrane, and then incubated overnight at 4°C with primary caspase antibodies (Cell signaling) and a phospho-mixed lineage kinase domain-like **(**MLKL) antibody (Cell signaling). The membrane was incubated with peroxidase-conjugated secondary antibodies at room temperature for 1 hour, and then proteins were visualized using enhanced chemiluminescence (Amersham, Piscataway, NJ). The blot was stripped with Blot Stripping Buffer (Thermo Fisher Scientific), and then re-probed with other antibodies as needed for the experiment.

### Statistical analysis

Data were expressed as mean ± standard deviation (SD). Student’s t-test or one-way analysis of variance (ANOVA) followed by Bonferroni’s post-hoc comparison tests was performed to compare groups using software IBM SPSS Statistics version 24. A p value <0.05 was considered significant.

## Results

### Identification of GADD45G binding proteins

**Fig 1:**
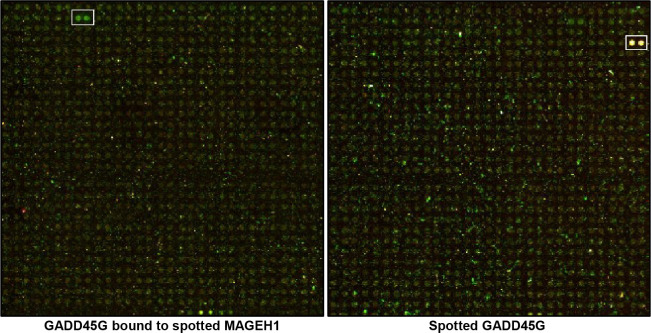
Identification of GADD45G interacting proteins using protein microarrays. The microarray was treated with in the order of an Alexa-Fluor 635 nm (red) conjugated anti-GADD45G antibody, GADD45G protein, and an Alexa-Fluor 532 nm (green) conjugated anti-GADD45G antibody. Therefore, a protein spotted on the microarray that binds to GADD45G protein should be captured by an Alexa-Fluor 532 nm conjugated anti-GADD45G antibody and emits green color. The left panel shows a protein (duplicate) that emits green color which was identified as MAGEH1 (NP_054780). In contrast, GADD45G protein originally spotted in the microarray should be captured by both anti-GADD45G antibodies (Alexa-Fluor 635 nm and Alexa-Fluor 532 nm) and emits yellow color that combines red and green color (right panel).

To identify functional networks of GADD45G proteins, more than 19,000 proteins were screened using human protein microarrays. One protein was found to bind to GADD45G, which was subsequently identified as MAGEH1 (Fig 1).

### GADD45G protein interacts with MAGEH1 protein

**Fig 2:**
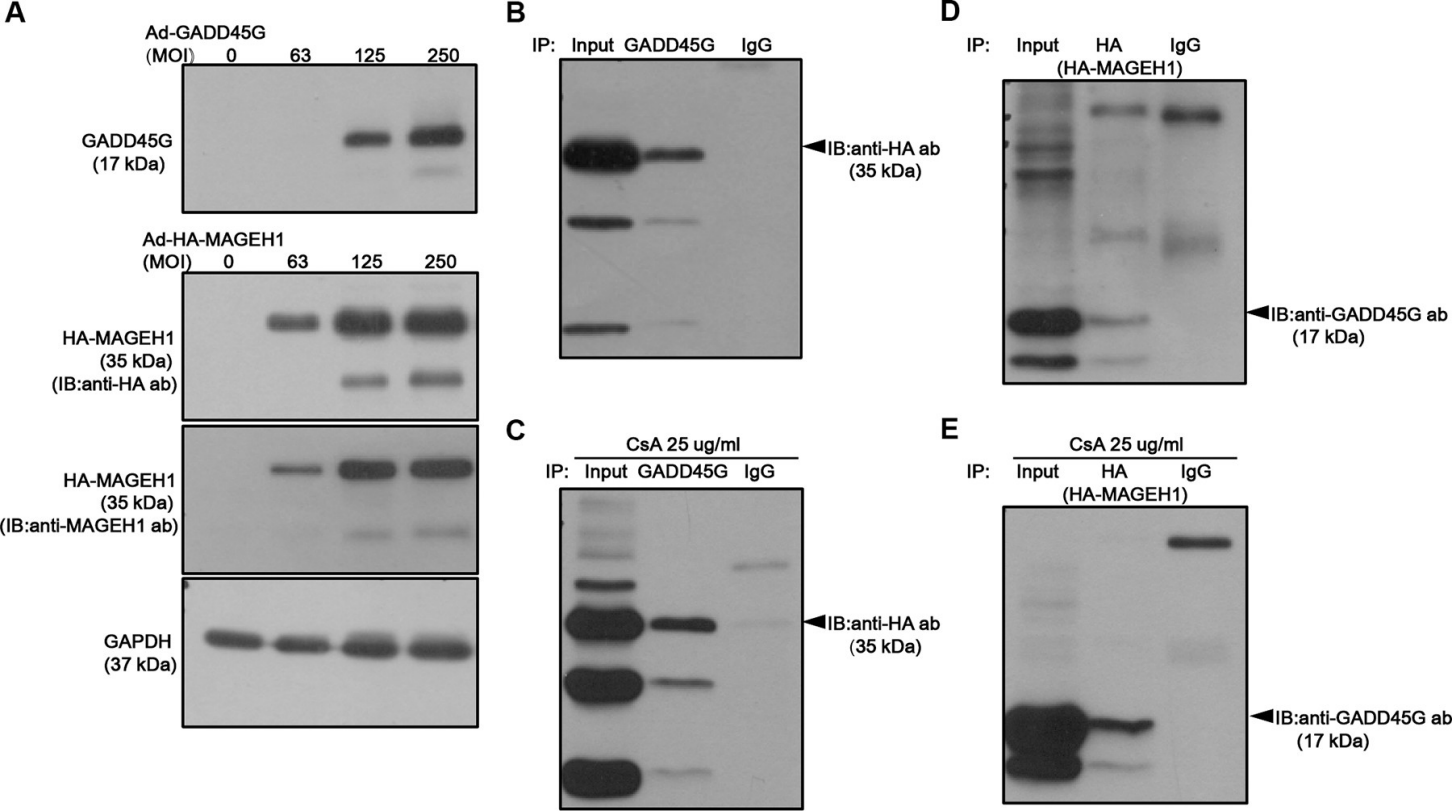
Co-immunoprecipitation to detect the interaction between GADD45G and MAGEH1. **(A)** Detection of overexpressed GADD45G and HA-MAGEH1 protein by Western blot. HRE cells were infected with a serial amount of Ad-GADD45G and Ad-HA-MAGEH1 for 24 hours. GADD45G proteins were able to be detected at an MOI of 125 and above. HA-MAGEH1 proteins were able to be detected at an MOI of 63 and above by both anti-HA and anti-MAGEH1 antibodies. **(B, C)** HRE cells were infected with Ad-GADD45G and Ad-HA-MAGEH1 simultaneously at an MOI of 250 in the presence or absence of 25 μg/ml CsA to harvest cellular proteins 24 hours later. Co-immunoprecipitation (IP) studies were performed using lysates prepared from HRE cells overexpressing GADD45G and HA-MAGEH1 proteins, which were immunoprecipitated with a monoclonal anti-GADD45G antibody and then immunoblotted (IB) using an anti-HA antibody. For controls, cell lysates were subjected to IP using control IgG. **(D, E)** In a reciprocal fashion, proteins were immunoprecipitated with anti-HA antibody and then immunoblotted using an anti-GADD45G antibody. Ad-GADD45G represents adenoviral vectors expressing GADD45G; Ad-HA-MAGEH1, adenoviral vectors expressing hemagglutinin (HA)-tagged MAGEH1 adenoviral vectors; MOI, multiplicity of infection.

**Fig 3:**
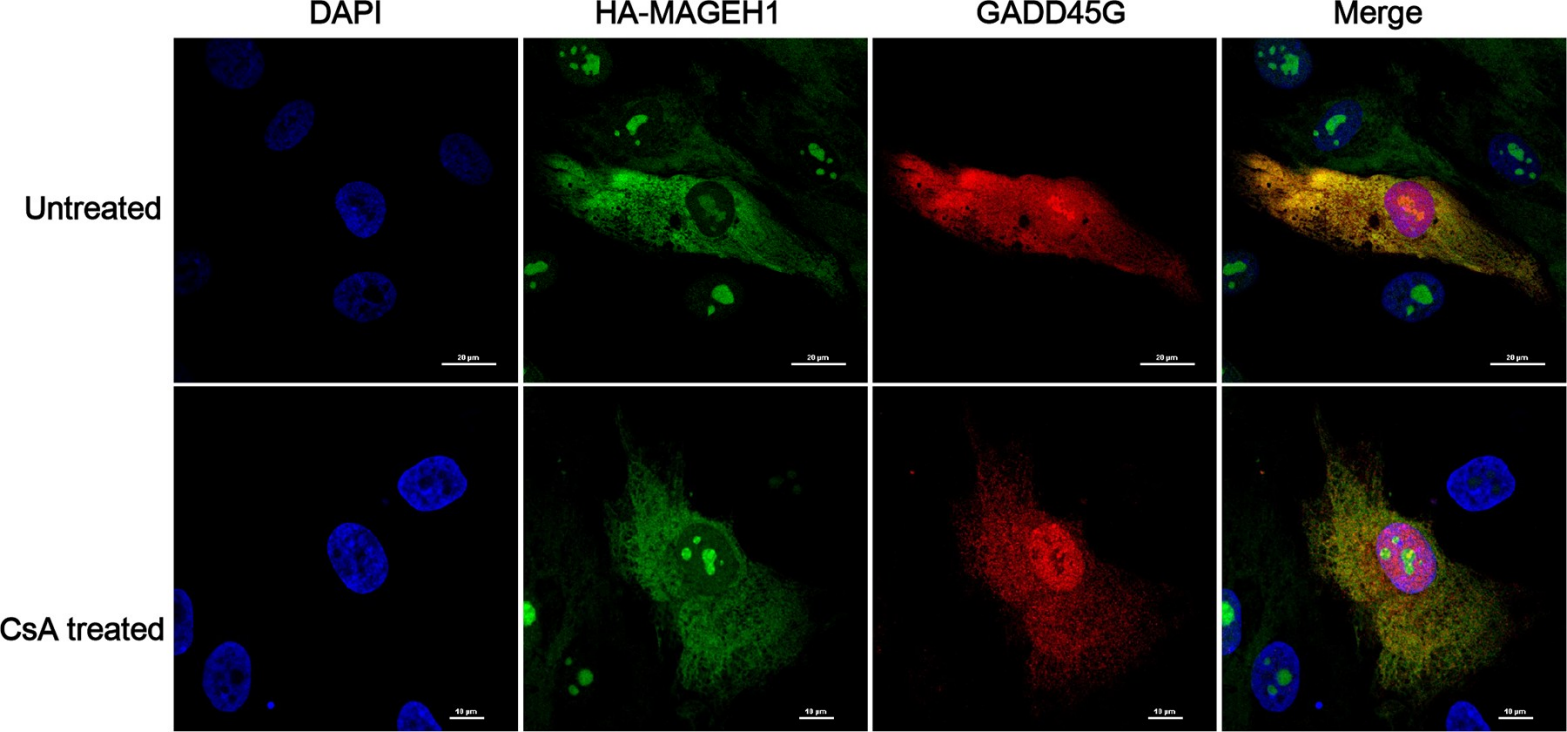
Confocal laser scanning immunofluorescence analysis to detect the interaction between GADD45G and MAGEH1. HRE cells were infected with Ad-GADD45G and Ad-HA-MAGEH1 simultaneously at an MOI of 250 for 24 hours. At the same time, HRE cells were not treated or treated with 25 μg/ml CsA for 24 hours and then subjected to immunofluorescent staining for the HA epitope (green) and GADD45G (red). In the merged image, yellow indicates costaining of the two proteins. DAPI staining (blue) was used to visualize the cell nucleus. The first row is showing cells not treated with CsA. The second row is showing cells treated with CsA. Scale bar: 10μm.

We performed co-immunoprecipitation assays to confirm the interaction between GADD45G and MAGEH1 proteins revealed by the protein microarray experiment. Since constitutive expressions of GADD45G and MAGEH1 proteins were too weak to be detected by western blotting, we overexpressed GADD45G and HA-MAGEH1 proteins in human renal epithelial (HRE) cells for co-immunoprecipitation assays in the presence or absence of CsA (Fig 2A). CsA is an immunosuppressive agent widely used in kidney transplantation to prevent acute rejection. However, CsA paradoxically can cause kidney injury [16,17] by inducing apoptosis of tubular and interstitial cells [18]. We found that immunoprecipitation of GADD45G co-precipitated with HA-MAGEH1 (Fig 2B and 2C) and conversely, immunoprecipitation of HA-MAGEH1 co-precipitated with GADD45G (Fig 2D and 2E), which confirms that GADD45G protein interacts with MAGEH1 protein. To further confirm such interaction, we performed immunofluorescence staining analysis. When MAGEH1 and GADD45G were coexpressed in HRE cells, the two proteins were colocalized at the cytoplasm of the cells as revealed by confocal microscopy (Fig 3), indicating possible direct interactions of MAGEH1 with GADD45G in HRE cells. The colocalization between the two proteins after CsA treatment was less evident probably due to poor cell condition from cytotoxic damage.

### *MAGEH1* gene expression is dependent on *GADD45G* gene expression

**Fig 4:**
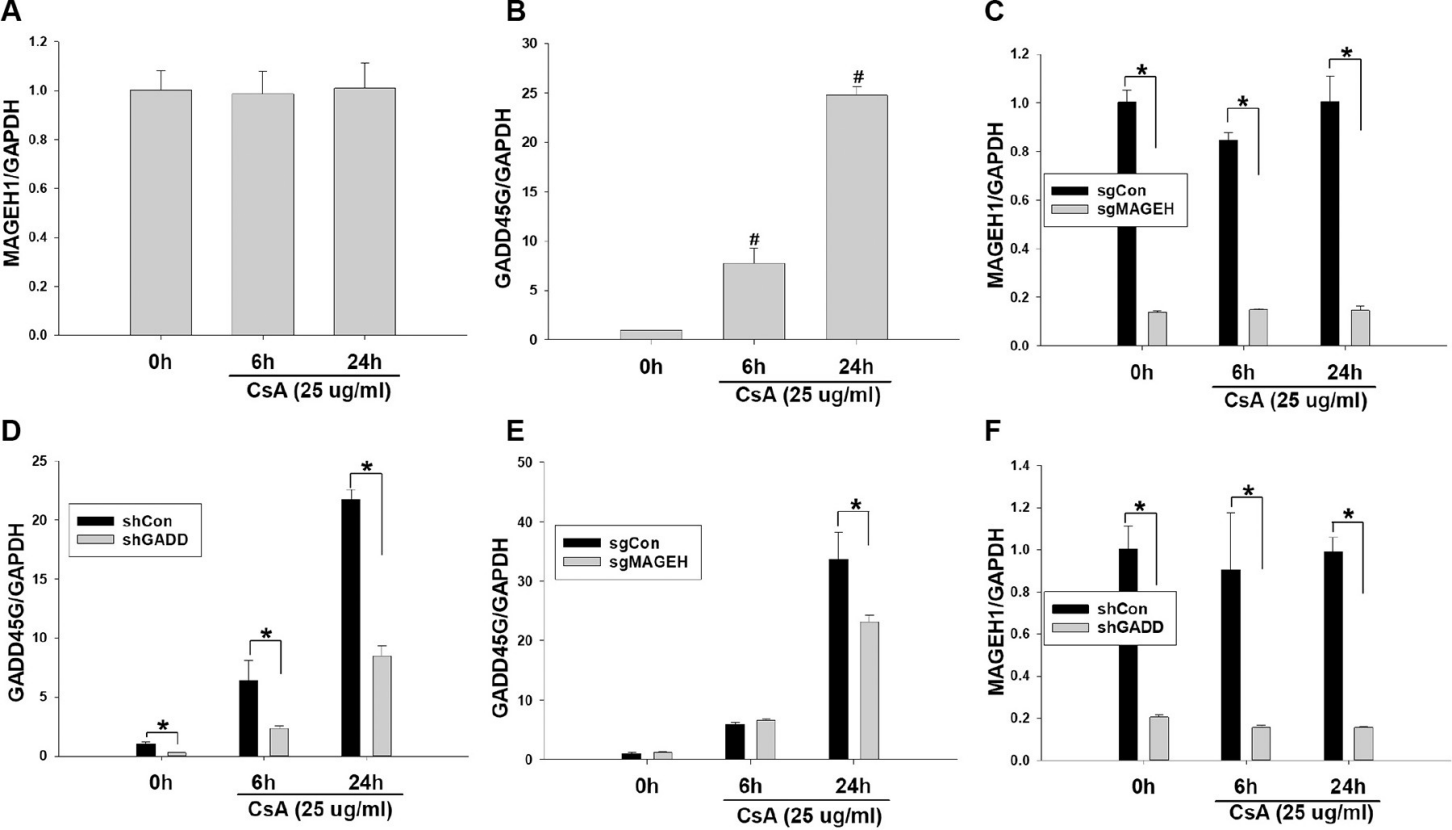
Quantitative real-time PCR for mRNA expression in CsA-treated HRE cells. **(A)** Time course of *MAGEH1* gene expression in response to CsA treatment. HRE cells were incubated with 25 μg/ml CsA for the indicated time. n = 3 each. **(B)** Time course of *GADD45G* gene expression in response to CsA treatment. HRE cells were incubated with 25 μg/ml CsA for the indicated time. n = 3 each. **(C)** Silencing of *MAGEH1* gene expression in sgMAGEH-HRE cells. n = 3 each**. (D)** Silencing of *GADD45G* gene expression in shGADD-HRE cells. n = 3 each**. (E)** Effect of silencing of *MAGEH1* on *GADD45G* expression in sgMAGEH-HRE cells. n = 3 each. **(F)** Effect of silencing of *GADD45*G on *MAGEH1* expression in shGADD-HRE cells. n = 3 each. Data were expressed as mean ± SD. One-way ANOVA followed by Bonferroni’s post-hoc comparisons tests (Panels A, B) or Student’s t-test (Panels C-F) was used to compare groups. A p value <0.05 was considered significant. #p<0.05 vs. all other groups; *p<0.05 between groups. GAPDH served as an internal control. sgMAGEH represents single guide RNA constructs to knockdown MAGEH1; sgCon, control single guide RNA constructs targeting no known genes; shGADD, short hairpin RNA constructs to knockdown GADD45G; shCon, control short hairpin RNA constructs targeting no known genes.

HRE cells were exposed to CsA to determine changes of gene expression of *GADD45G* and *MAGEH1* in response to nephrotoxic agents. Our results showed that *MAGEH1* mRNA was strongly expressed in HRE cells constitutively and that its expression was not affected by CsA (Fig 4A), whereas the level of *GADD45G* mRNA expression was very low constitutively and it was increased significantly in response to CsA treatment (Fig 4B). Given the interaction between GADD45G and MAGEH1 as shown above, we investigated their mutual influence using MAGEH1 (sgMAGEH-HRE) and GADD45G (shGADD-HRE) knockdown HRE cells. *MAGEH1* mRNA expression was downregulated by 82–85% in sgMAGEH-HRE cells compared to that in control HRE (sgCon-HRE) cells (Fig 4C). Similarly, *GADD45G* expression was downregulated by 61–63% in shGADD-HRE cells compared to that in control HRE (shCon-HRE) cells (Fig 4D). In sgMAGEH-HRE cells, *GADD45G* expression was decreased only to a small degree (Fig 4E), whereas *MAGEH1* expression was decreased by 82–84% in shGADD-HRE cells compared to that in control cells (Fig 4F). These data suggest that the expression of *MAGEH1* is largely dependent on the expression of *GADD45G*.

### CsA-induced renal tubular cell death is dependent on MAGEH1 and GADD45G expression

**Fig 5:**
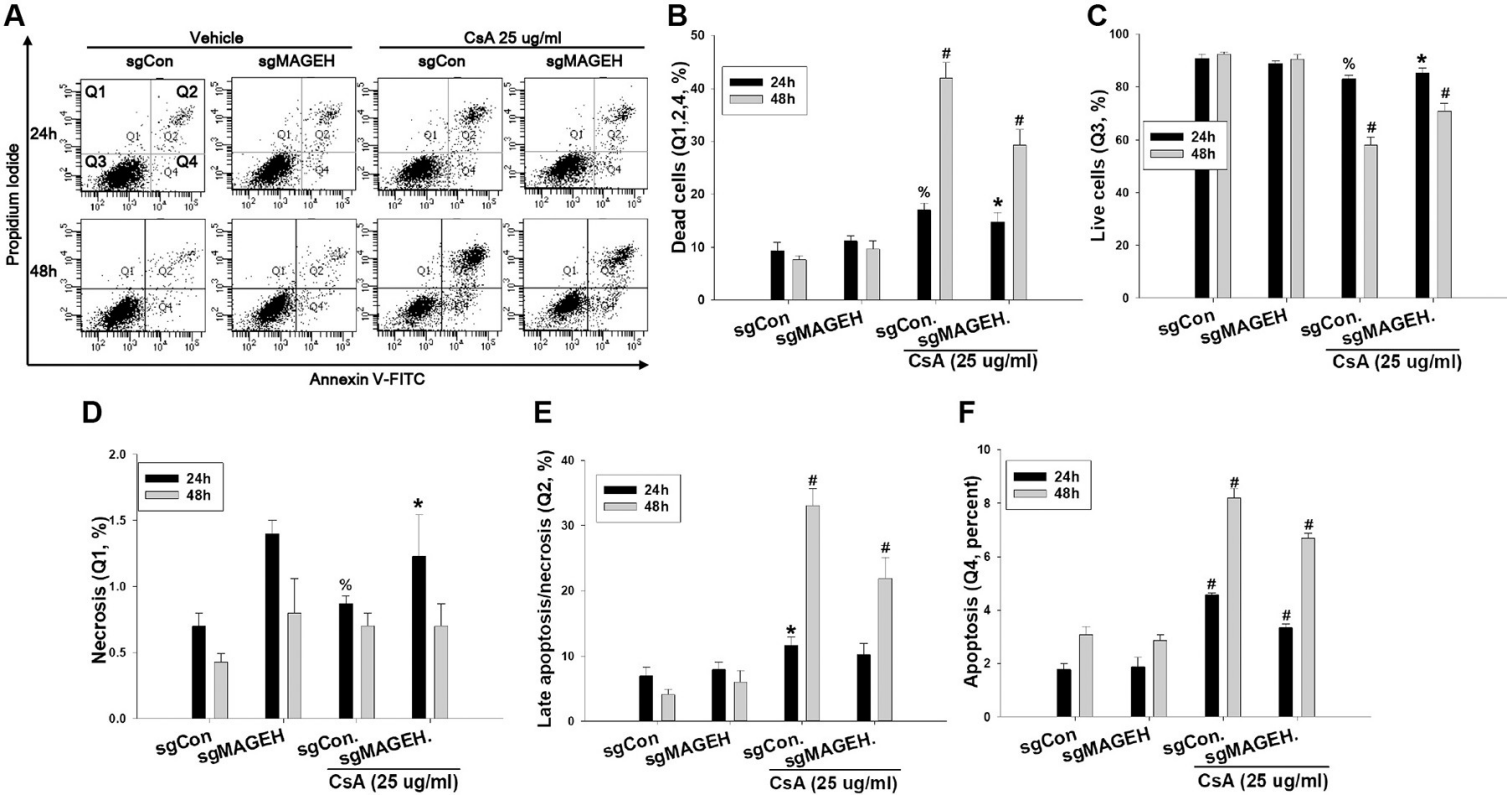
Quantification of apoptosis and necrosis by flow cytometry in CsA-treated HRE cells: Effect of MAGEH1 knockdown. sgMAGEH and sgCon stable HRE cells were incubated with 25 μg/ml CsA for the indicated time periods. **(A)** Representative scatter plots of flow cytometry. Quantification of **(B)** dead cells (Q1+Q2+Q4), **(C)** viable cells (PI negative and Annexin V negative, Q3), **(D)** necrotic/non-viable cells (PI positive and Annexin V negative, Q1) **(E)** late apoptotic/necrotic cells (PI positive and Annexin V positive, Q2), and **(F)** early apoptotic cells (PI negative and Annexin V positive, Q4). Each bar represents mean ± SD of 3 experiments. One-way ANOVA followed by Bonferroni’s post-hoc comparisons tests was used to compare groups. A p value <0.05 was considered significant. % p<0.05 vs. sgMAGEH; *p<0.05 vs. the corresponding sgCon group; #p<0.05 vs. all other corresponding groups. sgMAGEH represents single guide RNA constructs to knockdown MAGEH1; sgCon, control single guide RNA constructs targeting no known genes.

**Fig 6:**
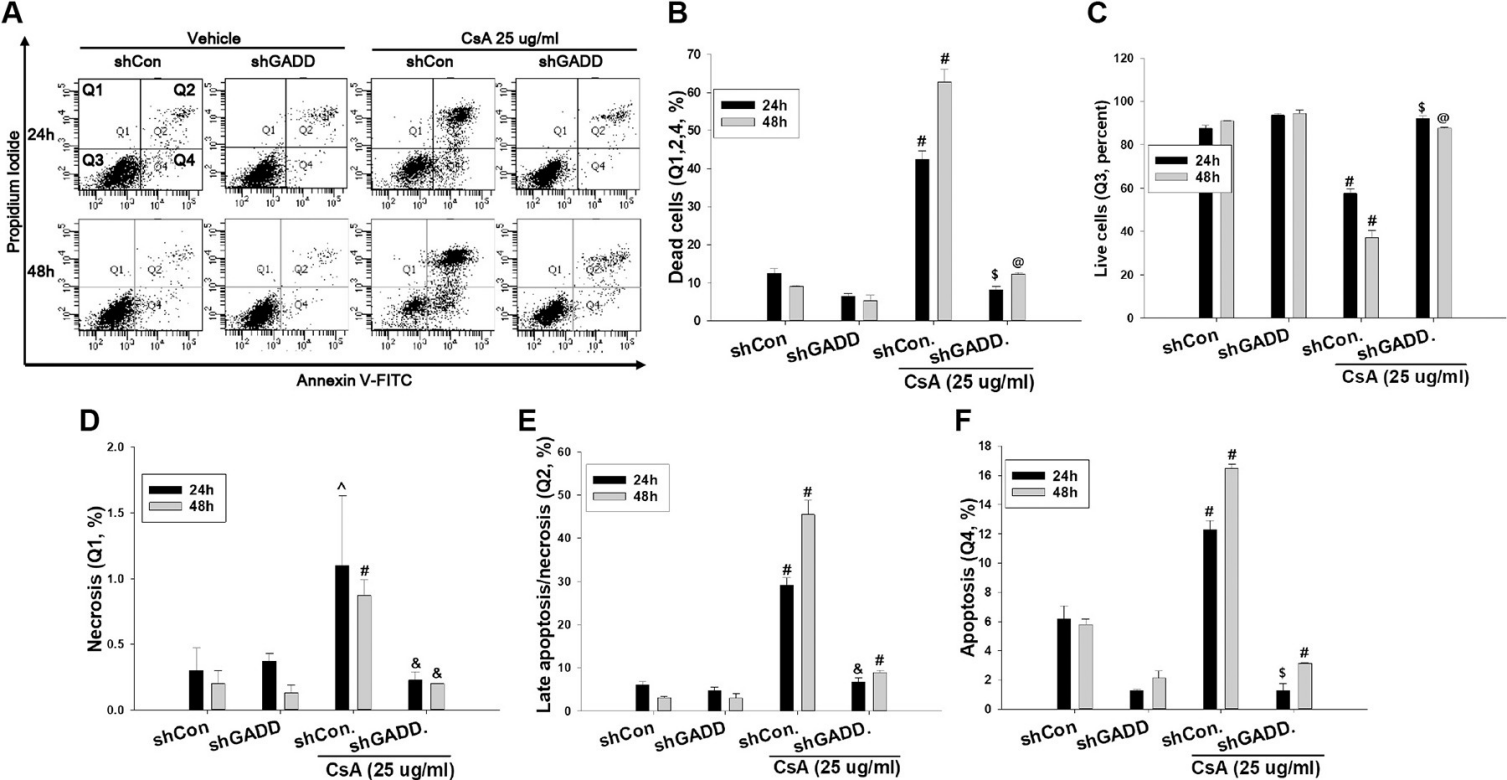
Quantification of apoptosis and necrosis by flow cytometry in CsA-treated HRE cells: Effect of GADD45G knockdown. shGADD and shCon stable HRE cells were treated with 25 μg/ml CsA for the indicated time periods. **(A)** Representative scatter plots of flow cytometry. Quantification of **(B)** dead cells (Q1+Q2+Q4), **(C)** viable cells (PI negative and Annexin V negative, Q3), **(D)** necrotic/non-viable cells (PI positive and Annexin V negative, Q1) **(E)** late apoptotic/necrotic cells (PI positive and Annexin V positive, Q2), and **(F)** early apoptotic cells (PI negative and Annexin V positive, Q4). Each bar represents mean ± SD of 3 experiments. One-way ANOVA followed by Bonferroni’s post-hoc comparisons tests was used to compare groups. A p value <0.05 was considered significant. #p<0.05 vs. all other corresponding groups; &p<0.05 vs. corresponding shCon/CsA groups; @p<0.05 vs. corresponding shGADD and shCon/CsA groups; $p<0.05 vs. corresponding shCon and shCon/CsA groups. shGADD represents shRNA constructs to knockdown GADD45G; shCon, control shRNA constructs targeting no known genes.

**Fig 7:**
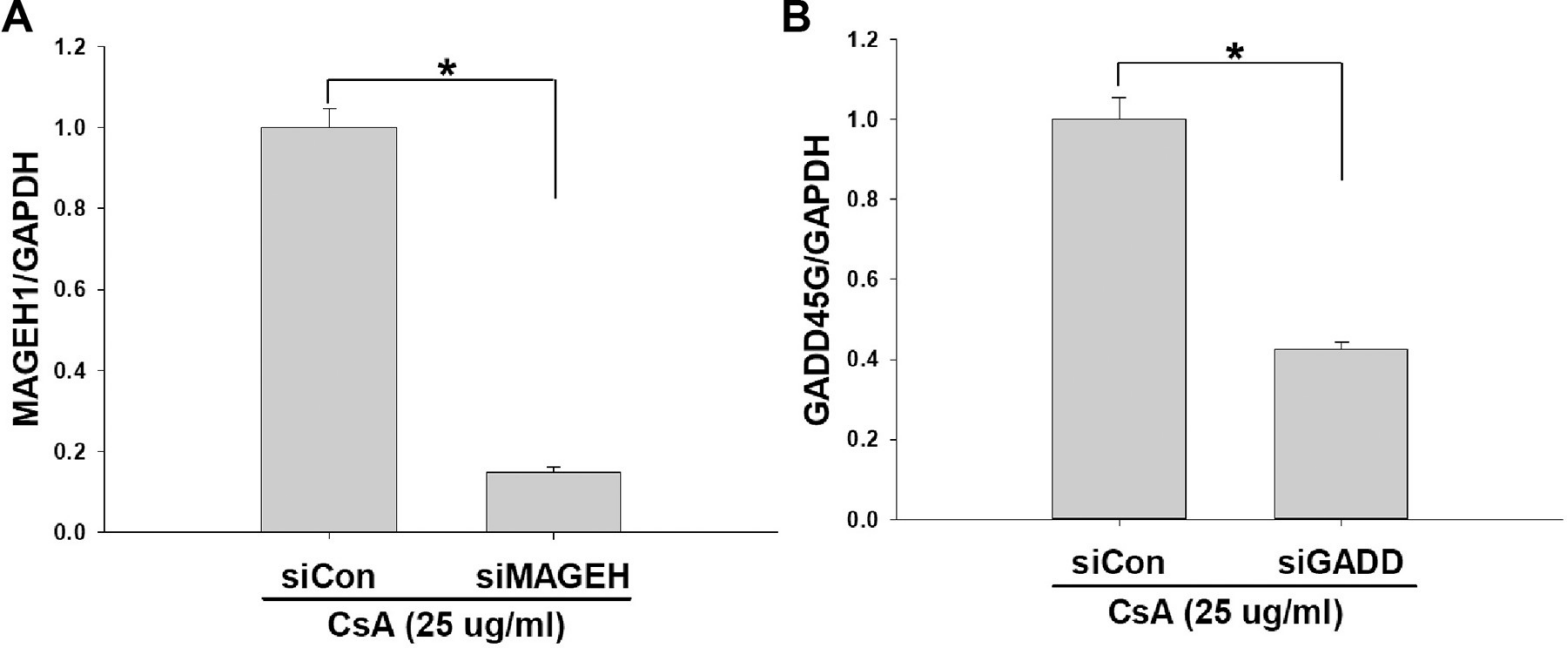
siRNA induced gene silencing in HK-2 cells. HK-2 cell line was transfected with siRNA for 24 h and then treated with 25 μg/ml CsA for 24 hours to measure mRNA expression using real-time PCR. **(A)** Transfection with MAGEH1 siRNA (siMAGEH) or control siRNA (siCon). n = 3 each. **(B)** Transfection with GADD45G siRNA (siGADD) or control siRNA (siCon). n = 3 each. GAPDH served as an internal control. Student’s t-test was used to compare groups. A p value <0.05 was considered significant. *p<0.05 between groups.

**Fig 8:**
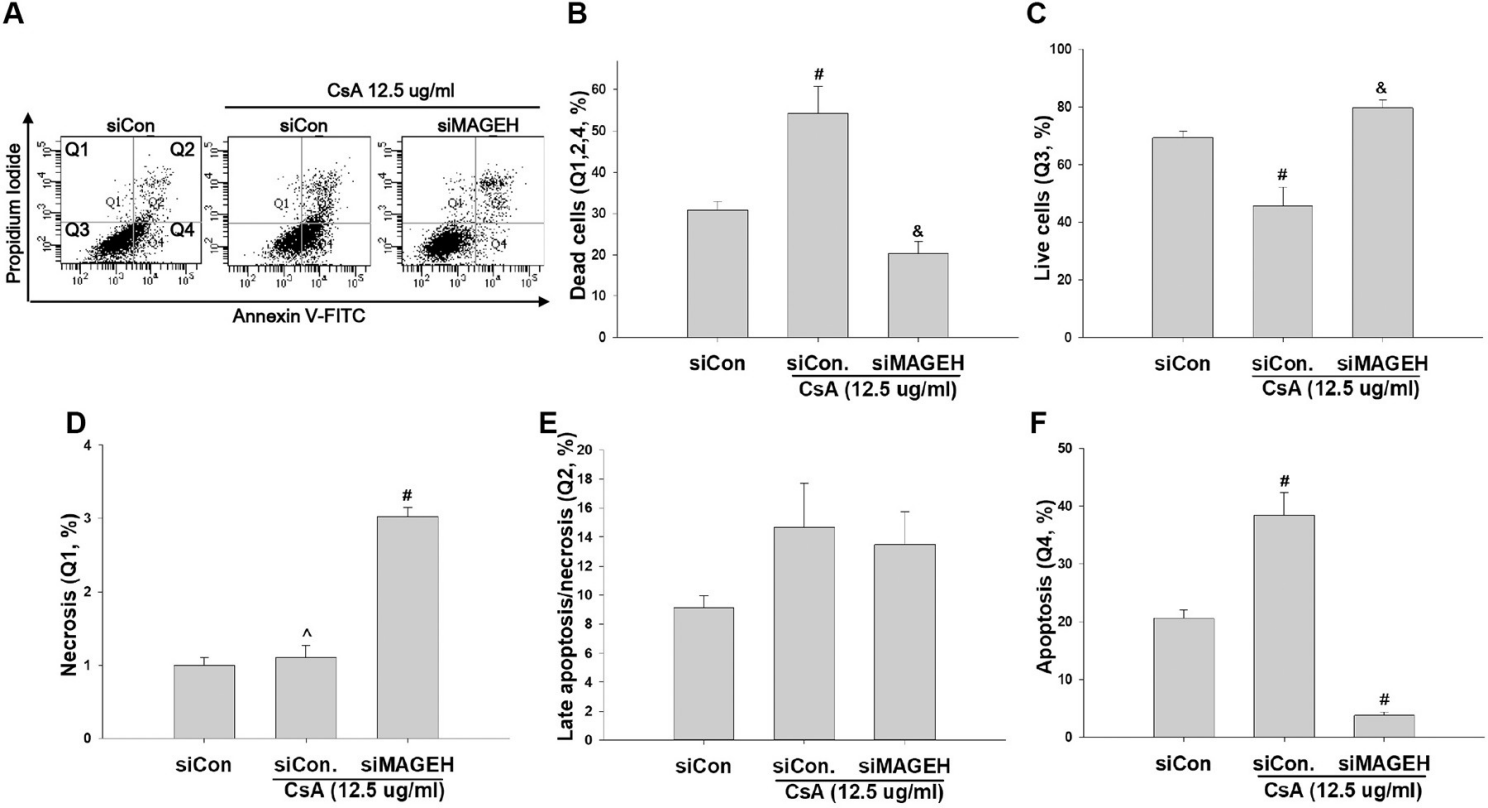
Quantification of cell apoptosis and survival by flow cytometry in CsA-treated HK-2 cells: Effect of MAGEH1 knockdown. HK-2 cell line was transfected with MAGEH1 siRNA or control siRNA. At 24 hours after transfection, cells were incubated with 12.5 μg/ml CsA for another 24 hours. **(A)** Representative scatter plots of flow cytometry. Quantification of **(B)** dead cells (Q1+Q2+Q4), **(C)** viable cells (PI negative and Annexin V negative, Q3), **(D)** necrotic/non-viable cells (PI positive and Annexin V negative, Q1) **(E)** late apoptotic/necrotic cells (PI positive and Annexin V positive, Q2), and **(F)** early apoptotic cells (PI negative and Annexin V positive, Q4). Each bar represents mean ± SD of 3 experiments. Statistical analysis was performed using One-way ANOVA followed by Bonferroni’s post-hoc comparisons tests. A p value <0.05 was considered significant. #p<0.05 vs. all other groups; &p<0.05 vs. siCon/CsA; ^p<0.05 vs. siMAGEH/CsA. siMAGEH stands for siRNA targeting MAGEH1; siCon, control siRNA targeting no known genes.

**Fig 9:**
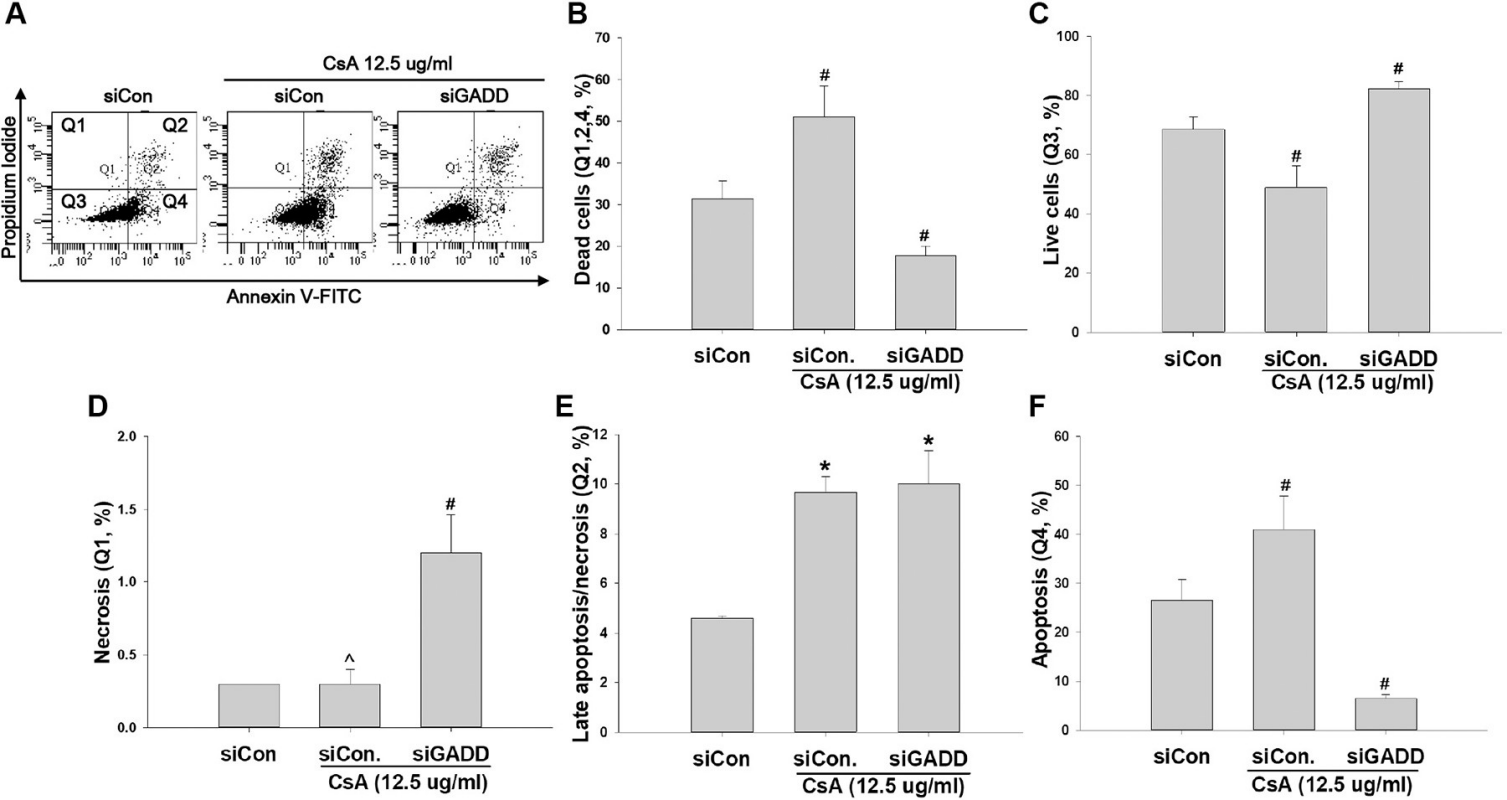
Quantification of cell apoptosis and survival by flow cytometry in CsA-treated HK-2 cells: Effect of GADD45G knockdown. HK-2 cell line was transfected with GADD45G siRNA or control siRNA. At 24 hours after transfection, cells were incubated with 12.5 μg/ml CsA for 24 another hours. **(A)** Representative scatter plots of flow cytometry. Quantification of **(B)** dead cells (Q1+Q2+Q4), **(C)** viable cells (PI negative and Annexin V negative, Q3), **(D)** necrotic/non-viable cells (PI positive and Annexin V negative, Q1) **(E)** late apoptotic/necrotic cells (PI positive and Annexin V positive, Q2), and **(F)** early apoptotic cells (PI negative and Annexin V positive, Q4). Each bar represents mean ± SD of 3 experiments. Statistical analysis was performed using One-way ANOVA followed by Bonferroni’s post-hoc comparisons tests. A p value <0.05 was considered significant. *p<0.05 vs. siCon; ^p<0.05 vs. siGADD/CsA; #p<0.05 vs. all other groups. siGADD stands for siRNA targeting GADD45G; siCon, control siRNA targeting no known genes.

To investigate the role of MAGEH1 in CsA-induced cell death, sgMAGEH-HRE cells were incubated with CsA, and then analyzed by flow cytometry. Results revealed that apoptosis (Q4) and necrosis (late apoptosis/necrosis, Q2) were significantly decreased by silencing of MAGEH1 in sgMAGEH-HRE cells, leading to a significantly increased survival of sgMAGEH-HRE cells compared to control sgCon-HRE cells (Q3) (Fig 5). In addition, we investigated the role of GADD45G in cell death using shGADD-HRE cells to confirm our previous findings [5], and to directly compare it with results of MAGEH1. Similar to results of sgMAGEH-HRE cells, apoptosis (Q4) and necrosis (late apoptosis/necrosis, Q2) were significantly decreased by silencing of GADD45G in shGADD-HRE cells, which led to a significantly increased survival of shGADD-HRE cells compared to control shCon-HRE cells (Q3) (Fig 6). To better understand roles of MAGEH1 and GADD45G in renal tubular cell death, we performed a subsequent experiment using different tubular cells and different gene silencing methods: In this experiment, we used the HK-2 cell line and gene silencing was induced by short interfering RNAs (siRNAs). Real-time PCR analysis revealed that MAGEH1 siRNA downregulated *MAGEH1* gene expression by 85% (Fig 7A) and GADD45G siRNA downregulated *GADD45G* gene expression by 58% in HK-2 cells (Fig 7B). To analyze the role of these genes in cell death, HK-2 cells were transfected with each siRNA followed by incubation with CsA, and then analyzed by flow cytometry. In accordance with the findings in HRE cells, CsA-induced apoptosis (Q4) was significantly decreased in MAGEH1 knockdown (siMAGEH) HK-2 cells, resulting in a significantly increased survival of siMAGEH HK-2 cells compared to control siRNA (siCon) transfected HK-2 cells (Q3) (Fig 8). Similarly, CsA-induced apoptosis (Q4) was significantly decreased in GADD45G knockdown (siGADD) HK-2 cells, resulting in a significantly increased survival of siGADD HK-2 cells compared to siCon transfected HK-2 cells (Q3) (Fig 9).

### Caspase activation is dependent on MAGEH1 and GADD45G expression

**Fig 10:**
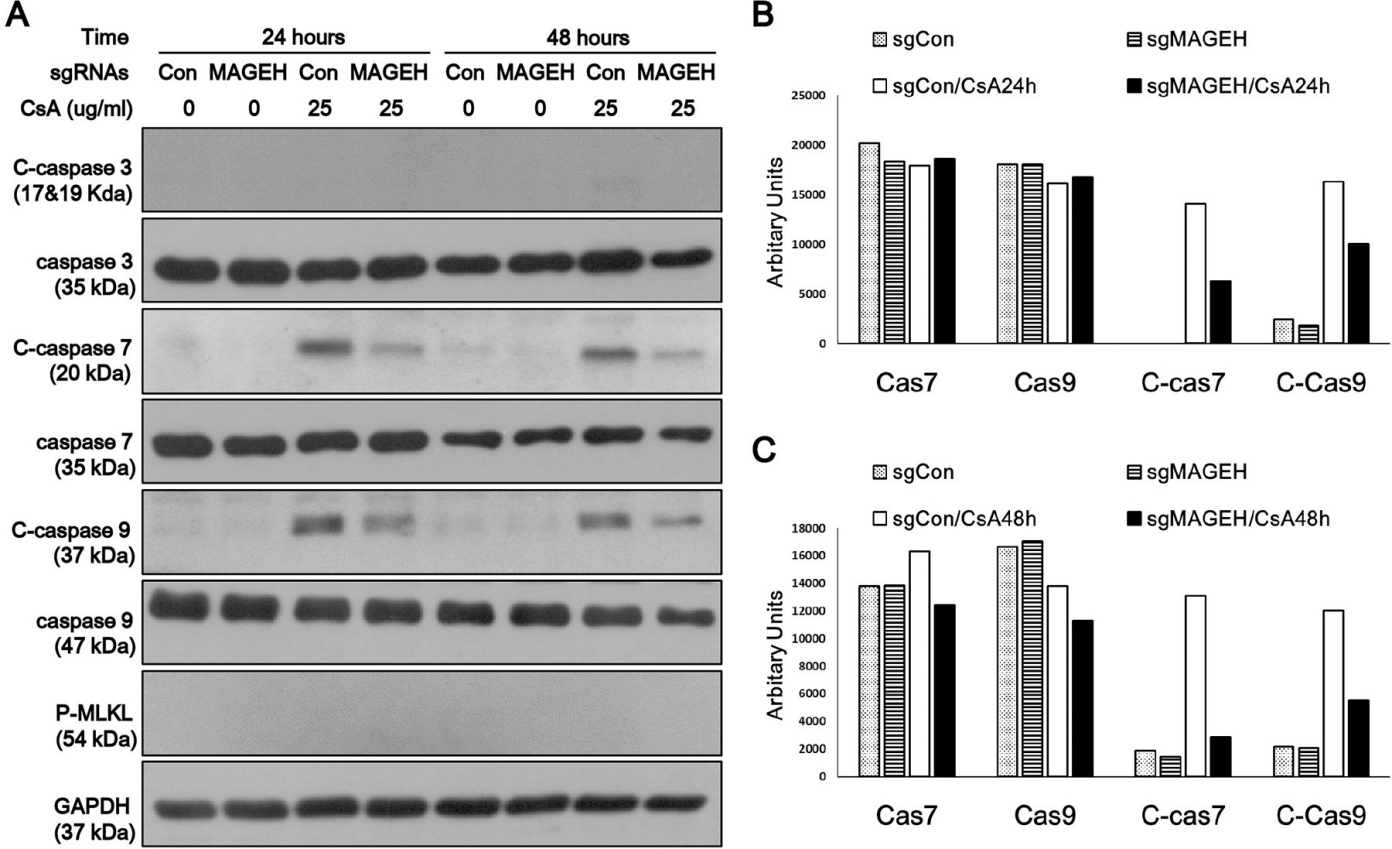
Western blot analysis of caspase activation in HRE cells: Effect of silencing of MAGEH1. sgMAGEH and sgCon HRE stable cells were incubated with 25 μg/ml CSA for 24 and 48 hours to harvest protein for western blotting **(A)**. Quantification of western blots (caspase 7, caspase 9, C-caspase 7, C-caspase 9) was performed using Image J at 24 hours **(B)**, and 48 hours of incubation with CsA **(C)**. C-caspases represent cleaved (active)-caspases; sgRNA MAGEH, single guide RNA constructs to knockdown MAGEH1; sgRNA Con, control single guide RNA constructs targeting no known genes.

**Fig 11:**
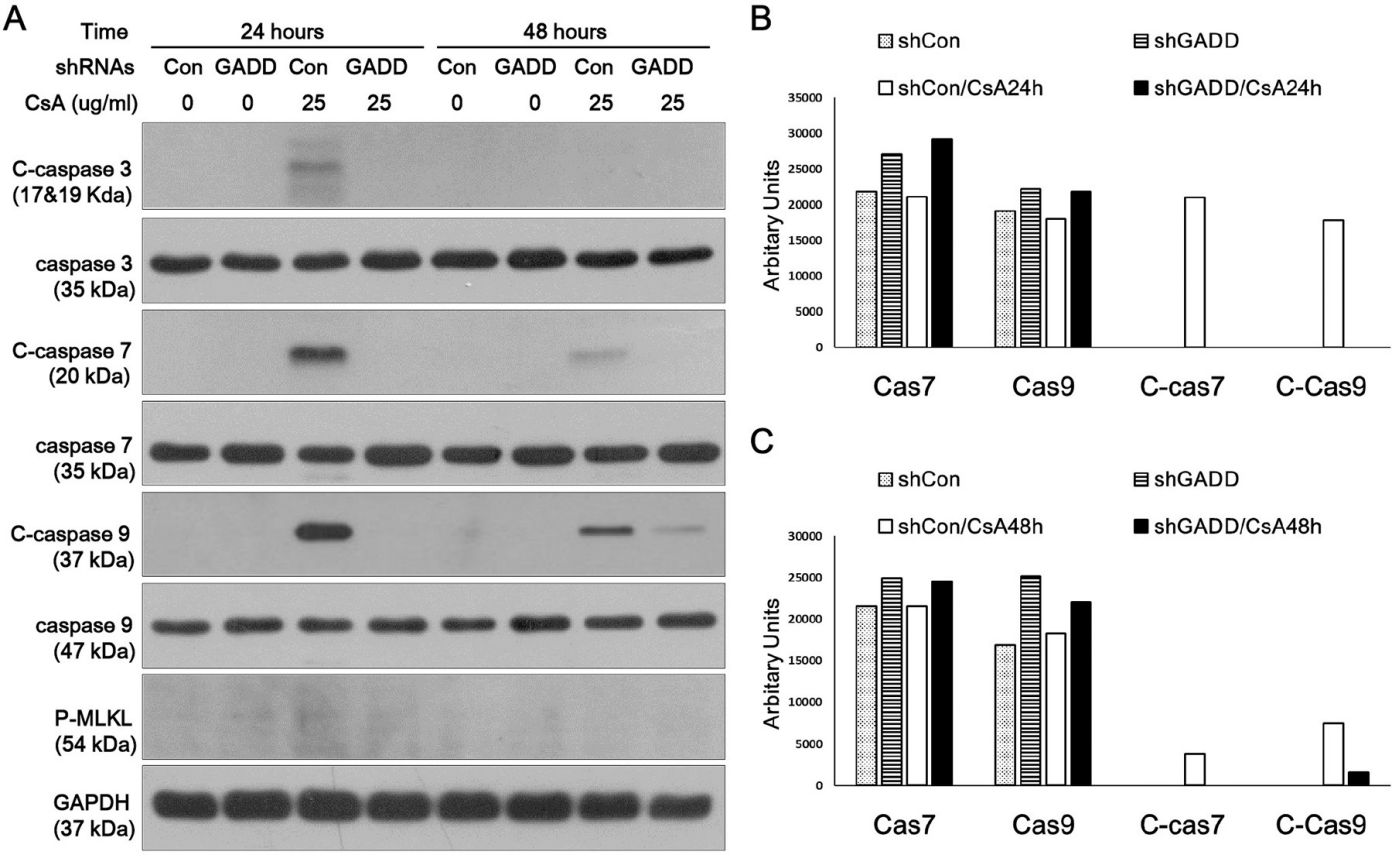
Western blot analysis of caspase activation in HRE cells: Effect of silencing of GADD45G. shGADD and shCon HRE stable cells were incubated with 25 μg/ml CSA for 24 and 48 hours to harvest protein for western blotting. **(A)**. Quantification of western blots (caspase 7, caspase 9, C-caspase 7, C-caspase 9) was performed using Image J at 24 hours **(B)**, and 48 hours of incubation with CsA **(C)**. C-caspases represent cleaved (active)-caspases; shRNA GADD, shRNA constructs to knockdown GADD45G; shRNA Con, control shRNA constructs targeting no known genes.

**Fig 12:**
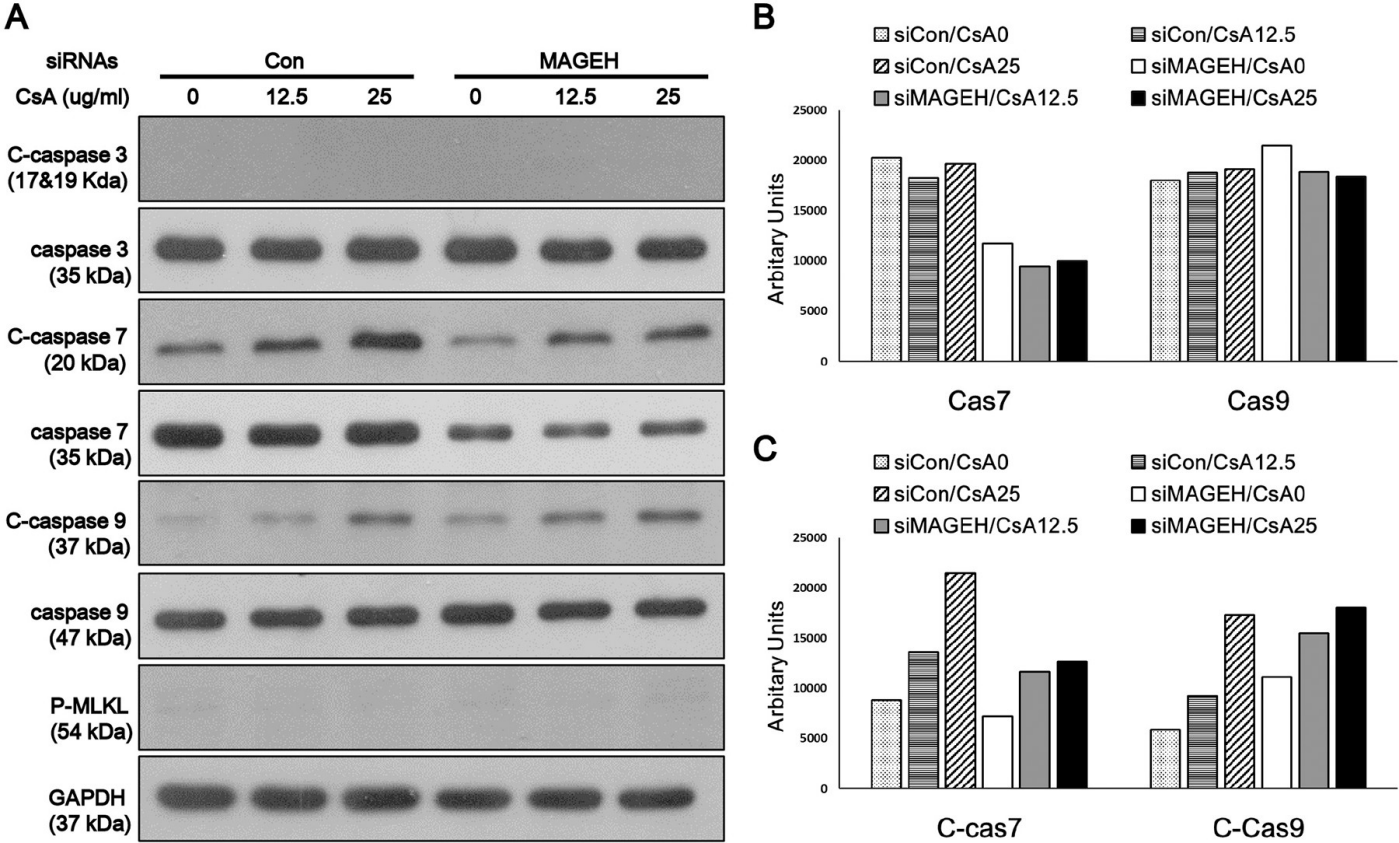
Western blot analysis of caspase activation in HK-2 cells: Effect of silencing of MAGEH1. HK-2 cells were transfected with siRNAs for 24 hours, and then treated with 12.5 μg/ml and 25 μg/ml CSA for 24 hours to harvest protein for western blotting **(A).** Quantification of western blots was performed using Image J for caspase 7 and caspase 9 **(B)**, and C-caspase 7 and C-caspase 9 **(C)**. C-caspases represent cleaved (active)-caspases; siRNA MAGEH, siRNA targeting MAGEH1; siRNA Con, control siRNA targeting no known genes.

**Fig 13:**
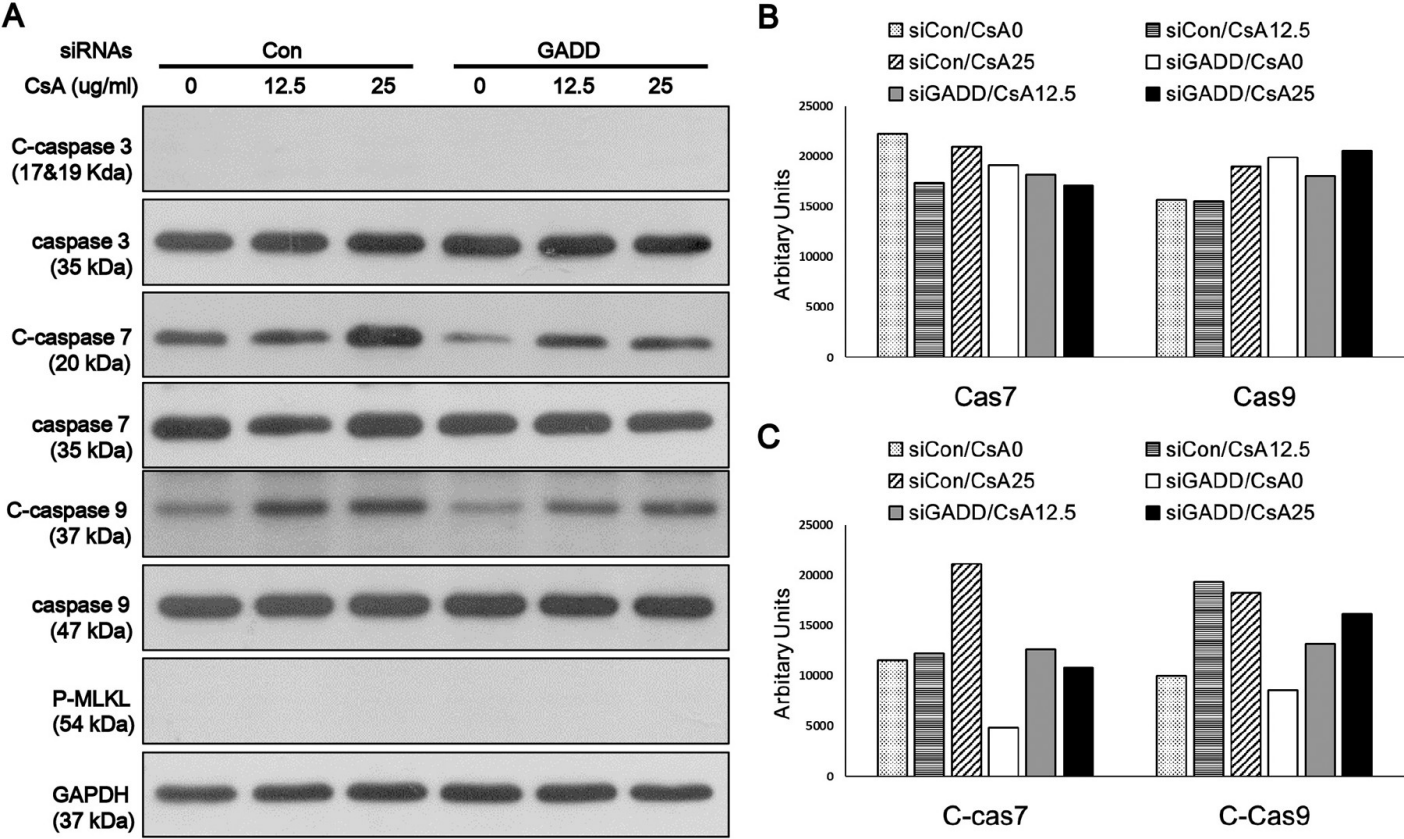
Western blot analysis of caspase activation in HK-2 cells: Effect of silencing of GADD45G. HK-2 cells were transfected with siRNAs for 24 hours, and then treated with 12.5 μg/ml and 25 μg/ml CSA for 24 hours to harvest protein for western blotting **(A).** Quantification of western blots was performed using Image J for caspase 7 and caspase 9 **(B)**, and C-caspase 7 and C-caspase 9 **(C)**. C-caspases represent cleaved (active)-caspases; siRNA GADD, siRNA targeting GADD45G; siRNA Con, control siRNA targeting no known genes.

To gain insight into the mechanism of MAGEH1-mediated regulation of cell death, we examined the expression of caspases which are well known mediators of apoptosis. MLKL, which is a key mediator of necroptosis (programmed necrosis), was also examined [19]. We found that caspase-7 and caspase-9 were activated by CsA as evidenced by increased cleaved forms in control sgCon-HRE cells, and such activation was prevented by silencing of MAGEH1 in sgMAGEH-HRE cells (Fig 10). MLKL was not activated by CsA indicating that necroptosis was not the pathway of cell death. In addition, we examined the role of GADD45G in caspase activation using shGADD-HRE cells to confirm our previous findings [5], and to directly compare it with results of MAGEH1. Similar to sgMAGEH-HRE cells, the activation of caspase-7 and caspase-9 in response to CsA treatment was inhibited by silencing of GADD45G in shGADD-HRE cells (Fig 11). To better understand the role of MAGEH1 and GADD45G in caspase activation, we performed a subsequent experiment using HK-2 cells where gene silencing was induced by siRNAs. In accordance with results in HRE cells, caspase-7 and caspase-9 were activated by CsA while caspase-7 activation was inhibited in siMAGEH HK-2 cells. Notably, total caspase-7 was also decreased in siMAGEH HK-2 cells. Unlike in HRE cells, however, the activation of caspase-9 was not inhibited in siMAGEH HK-2 cells (Fig 12). These data suggest that caspase-7 is an invariable downstream target of MAGEH1 across different types of renal tubular cells. In siGADD HK-2 cells, activation of caspase-7 and -9 was prevented by silencing of GADD45G in accordance with results in shGADD-HRE cells (Fig 13).

## Discussion

We have previously shown that GADD45G is involved in caspase dependent apoptosis of human renal tubular cells [5]. To further elucidate the mechanistic detail about how GADD45G functions in apoptosis, we screened its binding proteins using a protein microarray and we were able to find one such molecule, which was subsequently identified as MAGEH1, a member of type II MAGE proteins. To confirm this finding, we performed co-immunoprecipitation assay which provided us a clear-cut evidence that GADD45G interacts with MAGEH1. Despite such interaction, both molecules were found to respond to cytotoxic stimuli in different ways: MAGEH1 gene expression was not altered by CsA whereas GADD45G gene showed a significant increase in response to CsA treatment. In addition, MAGEH1 gene expression was significantly decreased upon CsA treatment when GADD45G gene expression was silenced indicating that the expression of MAGEH1 was dependent on the expression of GADD45G. It is likely that GADD45G is one of the upstream molecules that regulates transcription of MAGEH1 in a way to facilitate the apoptotic process of damaged cells. Based on these results, we hypothesize that GADD45G expression increases in response to cytotoxic stimuli, which then prevents the decrease of its binding protein, MAGEH1, leading to an increase of the formation of GADD45G and MAGEH1 complexes.

While there have not been many studies linking MAGEH1 to renal disorders, a few studies have provided evidence that MAGEH1 may play a critical role in the pathogenesis of renal diseases [13,14]. In the present study, we explored the role of MAGEH1 in nephrotoxic injury and found that MAGEH1 is involved in the induction of apoptosis of renal tubular cells. In fact, several reports have indicated that MAGE proteins may play a role in inducing apoptosis: In neuronal cells, it was shown that MAGED1 promotes apoptosis [20]. In addition, MAGEA4 was shown to promote apoptotic cell death induced by chemotherapeutic agents in lung cancer cells [21]. In contrast, inhibitory effect of MAGEs on apoptosis has also been reported: MAGEA3 was shown to promote proliferation while suppressing apoptosis of cervical cancer cells [22]. Similarly, it was shown that MAGEA3 suppresses apoptosis of multiple myeloma cells [23], and MAGE-C suppresses apoptosis of melanoma and colon cancer cell lines [24]. These reports collectively indicate that each member of MAGEs may play a different role in apoptosis.

We have previously shown that GADD45G is essential for CsA-induced activation of caspases [5]. Here, we extended our studies by demonstrating that GADD45G binds to MAGEH1 and that MAGEH1 is also essential for such activation of caspases. It has been well accepted that caspases play a critical role in regulating apoptosis: The intrinsic pathway of apoptotic signaling cascades is triggered by DNA-damage agents, releasing cytochrome c into the cytosol, which is associated with Apaf-1 to form the apoptosome. The apoptosome then activates caspase-9, an initiator caspase, which then activates executioner caspase-3 and caspase-7, ultimately inducing apoptosis [25]. Our study showed that silencing of MAGEH1 inhibited the activation of caspase-7 and caspase-9 in HRE cells, whereas it inhibited the activation of capase-7 but not caspase-9 in HK-2 cells. On the other hand, silencing of GADD45G was shown to inhibit the activation of both caspase-7 and caspase-9 in HK-2 cells as well as in HRE cells. In addition, silencing of MAGEH1 decreased the total caspase-7 level in HK-2 cells but not in HRE cells. It is unknown at present why the pattern of MAGEH1-dependent caspase expression or its activation was different in HK-2 cells from that in HRE cells. However, at least it is evident from our data that caspase-7 is an invariable downstream target of MAGEH1 in both HRE and HK-2 cells as well as through both types of gene silencing (stable vs. transient). Further studies are needed to elucidate the mechanistic details about how MAGEH1 and GADD45G play a role in the caspase-dependent apoptotic pathway.

In conclusion, we propose a novel pathway of apoptosis of renal tubular cells in response to nephrotoxic stimuli where MAGEH1 and GADD45G are implicated. We hope our research will contribute to the understanding of mechanisms for the development of renal tubular cell death leading to acute kidney injury. Increased knowledge of these mechanisms might provide a basis to find new therapeutic targets for acute kidney injury.

## Supporting information

S1 Data Protein microarray (HuProt human protein microarray version 2). (XLSX)

S1 Raw images Images of all original blot and gel images. (PDF)

## References

[1] Smith ML, Chen IT, Zhan Q, Bae I, Chen CY, Gilmer TM, et al. Interaction of the p53-regulated protein Gadd45 with proliferating cell nuclear antigen. Science. 1994;266(5189):1376–80. Epub 1994/11/25. doi: 10.1126/science.7973727 . 7973727

[2] Zerbini LF, Wang Y, Czibere A, Correa RG, Cho JY, Ijiri K, et al. NF-kappa B-mediated repression of growth arrest- and DNA-damage-inducible proteins 45alpha and gamma is essential for cancer cell survival. Proc Natl Acad Sci U S A. 2004;101(37):13618–23. Epub 2004/09/09. doi: 10.1073/pnas.0402069101 ; PubMed Central PMCID: PMC518803. 15353598 PMC518803

[3] Shin GT, Kim DR, Lim JE, Yim H, Kim H. Upregulation and function of GADD45gamma in unilateral ureteral obstruction. Kidney Int. 2008;73(11):1251–65. Epub 2008/03/21. doi: 10.1038/ki.2008.93 . 18354378

[4] Yu S, Cho J, Park I, Kim SJ, Kim H, Shin GT. Urinary GADD45gamma expression is associated with progression of lgA nephropathy. Am J Nephrol. 2009;30(2):135–9. Epub 2009/03/19. doi: 10.1159/000209317 . 19293565

[5] Shin GT, Lee HJ, Park JE. Growth arrest and DNA damage 45gamma is required for caspase-dependent renal tubular cell apoptosis. PLoS One. 2019;14(2):e0212818. Epub 2019/02/23. doi: 10.1371/journal.pone.0212818 ; PubMed Central PMCID: PMC6386268. 30794682 PMC6386268

[6] van der Bruggen P, Traversari C, Chomez P, Lurquin C, De Plaen E, Van den Eynde B, et al. A gene encoding an antigen recognized by cytolytic T lymphocytes on a human melanoma. Science. 1991;254(5038):1643–7. Epub 1991/12/13. doi: 10.1126/science.1840703 . 1840703

[7] Rogner UC, Wilke K, Steck E, Korn B, Poustka A. The melanoma antigen gene (MAGE) family is clustered in the chromosomal band Xq28. Genomics. 1995;29(3):725–31. Epub 1995/10/10. doi: 10.1006/geno.1995.9945 . 8575766

[8] Lurquin C, De Smet C, Brasseur F, Muscatelli F, Martelange V, De Plaen E, et al. Two members of the human MAGEB gene family located in Xp21.3 are expressed in tumors of various histological origins. Genomics. 1997;46(3):397–408. Epub 1998/01/27. doi: 10.1006/geno.1997.5052 . 9441743

[9] Lucas S, De Plaen E, Boon T. MAGE-B5, MAGE-B6, MAGE-C2, and MAGE-C3: four new members of the MAGE family with tumor-specific expression. Int J Cancer. 2000;87(1):55–60. Epub 2000/06/22. . 10861452

[10] Gillespie AM, Coleman RE. The potential of melanoma antigen expression in cancer therapy. Cancer Treat Rev. 1999;25(4):219–27. Epub 1999/08/17. doi: 10.1053/ctrv.1999.0126 . 10448130

[11] Chomez P, De Backer O, Bertrand M, De Plaen E, Boon T, Lucas S. An overview of the MAGE gene family with the identification of all human members of the family. Cancer Res. 2001;61(14):5544–51. Epub 2001/07/17. . 11454705

[12] Florke Gee RR, Chen H, Lee AK, Daly CA, Wilander BA, Fon Tacer K, et al. Emerging roles of the MAGE protein family in stress response pathways. J Biol Chem. 2020;295(47):16121–55. Epub 2020/09/15. doi: 10.1074/jbc.REV120.008029 ; PubMed Central PMCID: PMC7681028. 32921631 PMC7681028

[13] Laghmani K, Beck BB, Yang SS, Seaayfan E, Wenzel A, Reusch B, et al. Polyhydramnios, Transient Antenatal Bartter’s Syndrome, and MAGED2 Mutations. N Engl J Med. 2016;374(19):1853–63. Epub 2016/04/28. doi: 10.1056/NEJMoa1507629 . 27120771

[14] Valino-Rivas L, Cuarental L, Agustin M, Husi H, Cannata-Ortiz P, Sanz AB, et al. MAGE genes in the kidney: identification of MAGED2 as upregulated during kidney injury and in stressed tubular cells. Nephrol Dial Transplant. 2019;34(9):1498–507. Epub 2018/12/13. doi: 10.1093/ndt/gfy367 . 30541139

[15] Livak KJ, Schmittgen TD. Analysis of relative gene expression data using real-time quantitative PCR and the 2(-Delta Delta C(T)) Method. Methods. 2001;25(4):402–8. Epub 2002/02/16. doi: 10.1006/meth.2001.1262 . 11846609

[16] Burdmann EA, Andoh TF, Yu L, Bennett WM. Cyclosporine nephrotoxicity. Seminars in Nephrology. 2003;23(5):465–76. doi: 10.1016/s0270-9295(03)00090-1 13680536

[17] Kopp JB, Klotman PE. Cellular and molecular mechanisms of cyclosporin nephrotoxicity. J Am Soc Nephrol. 1990;1(2):162–79. doi: 10.1681/ASN.V12162 2104260

[18] Thomas SE, Andoh TF, Pichler RH, Shankland SJ, Couser WG, Bennett WM, et al. Accelerated apoptosis characterizes cyclosporine-associated interstitial fibrosis. Kidney international. 1998;53(4):897–908. doi: 10.1111/j.1523-1755.1998.00835.x . 9551396

[19] Sun L, Wang H, Wang Z, He S, Chen S, Liao D, et al. Mixed lineage kinase domain-like protein mediates necrosis signaling downstream of RIP3 kinase. Cell. 2012;148(1–2):213–27. Epub 2012/01/24. doi: 10.1016/j.cell.2011.11.031 . 22265413

[20] Salehi AH, Roux PP, Kubu CJ, Zeindler C, Bhakar A, Tannis LL, et al. NRAGE, a novel MAGE protein, interacts with the p75 neurotrophin receptor and facilitates nerve growth factor-dependent apoptosis. Neuron. 2000;27(2):279–88. Epub 2000/09/14. doi: 10.1016/s0896-6273(00)00036-2 . 10985348

[21] Peikert T, Specks U, Farver C, Erzurum SC, Comhair SA. Melanoma antigen A4 is expressed in non-small cell lung cancers and promotes apoptosis. Cancer Res. 2006;66(9):4693–700. Epub 2006/05/03. doi: 10.1158/0008-5472.CAN-05-3327 . 16651421

[22] Gao X, Li Q, Chen G, He H, Ma Y. MAGEA3 promotes proliferation and suppresses apoptosis in cervical cancer cells by inhibiting the KAP1/p53 signaling pathway. Am J Transl Res. 2020;12(7):3596–612. Epub 2020/08/11. ; PubMed Central PMCID: PMC7407682. 32774721 PMC7407682

[23] Mei AH, Tung K, Han J, Perumal D, Lagana A, Keats J, et al. MAGE-A inhibit apoptosis and promote proliferation in multiple myeloma through regulation of BIM and p21(Cip1). Oncotarget. 2020;11(7):727–39. Epub 2020/03/07. doi: 10.18632/oncotarget.27488 ; PubMed Central PMCID: PMC7041939. 32133047 PMC7041939

[24] Yang B, O’Herrin SM, Wu J, Reagan-Shaw S, Ma Y, Bhat KM, et al. MAGE-A, mMage-b, and MAGE-C proteins form complexes with KAP1 and suppress p53-dependent apoptosis in MAGE-positive cell lines. Cancer Res. 2007;67(20):9954–62. Epub 2007/10/19. doi: 10.1158/0008-5472.CAN-07-1478 . 17942928

[25] Lamkanfi M, Kanneganti TD. Caspase-7: a protease involved in apoptosis and inflammation. Int J Biochem Cell Biol. 2010;42(1):21–4. Epub 2009/09/29. doi: 10.1016/j.biocel.2009.09.013 ; PubMed Central PMCID: PMC2787741. 19782763 PMC2787741

