# Supplementary material for: MAGEH1 interacts with GADD45G and induces renal tubular cell apoptosis
Source: PLoS One. 2021 Nov 17;16(11):e0260135. doi: 10.1371/journal.pone.0260135 (PMC8598065; doi:10.1371/journal.pone.0260135)
Supplement: S1 Raw images — (PDF) [file pone.0260135.s002.pdf]

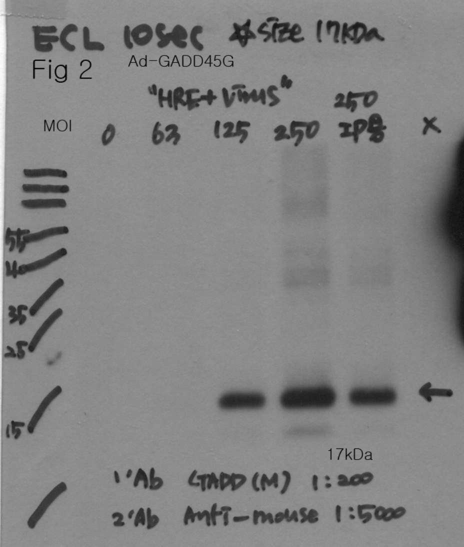
ECL 10sec ☆size 17kDa
Ad-GADD45G
"HRE+Virus"
MOI
0
63
125
250
250 IP
x
55
40
35
25
15
17kDa
1'Ab GADD(M) 1:200
2'Ab Anti-mouse 1:5000
Fig 2

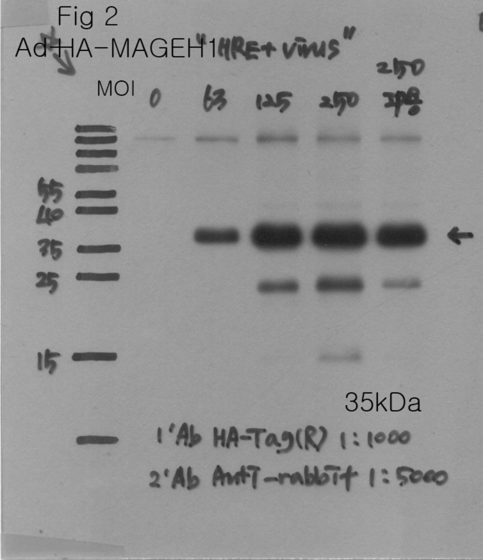
Fig 2
Ad-HA-MAGEH1
"HRE+ virus"
MOI
0
63
125
250
55
40
35
25
15
35kDa
1'Ab HA-Tag(R) 1:1000
2'Ab Anti-rabbit 1:5000

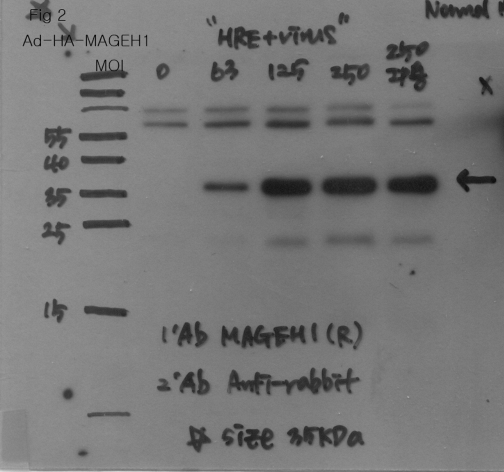
Fig 2
Ad-HA-MAGEH1
MOI
"HRE+virus"
0
63
125
250
250
55
40
35
25
15
1'Ab MAGEH1 (R)
2'Ab Anti-rabbit
size 35KDa

Fig 2

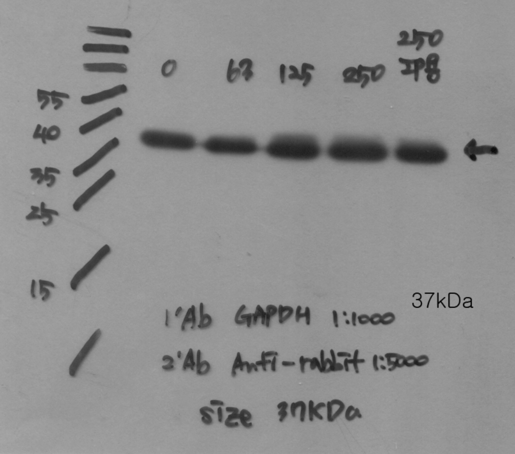
0
63
125
250
250
55
40
35
25
15
37kDa
1'Ab GAPDH 1:1000
2'Ab Anti-rabbit 1:5000
size 37KDa

Fig 2

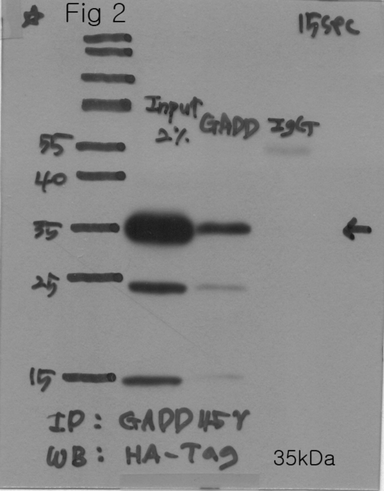

35kDa

Fig 2

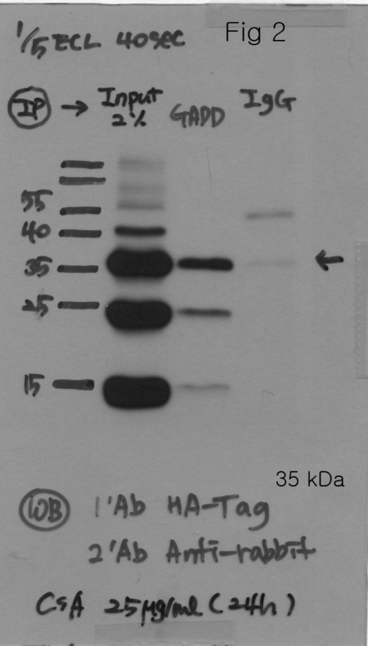
1/5 ECL 40sec
IP → Input 2% GADD IgG
55
40
35
25
15
WB 1'Ab HA-Tag
2'Ab Anti-rabbit
CsA 25μg/ml (24h)

35 kDa

Fig 2

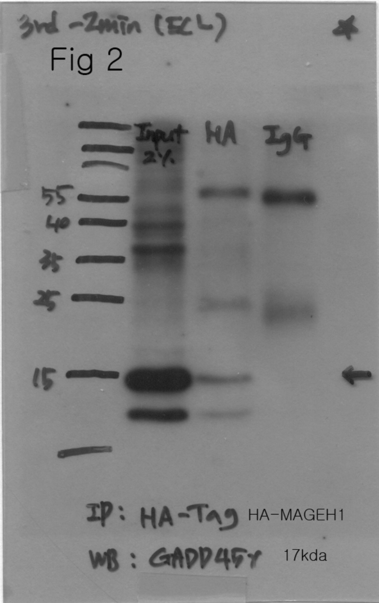
3rd -2min (ECL)
Input 2%
HA
IgG
55
40
35
25
15
IP : HA-Tag
HA-MAGEH1
WB : GADD45γ
17kda

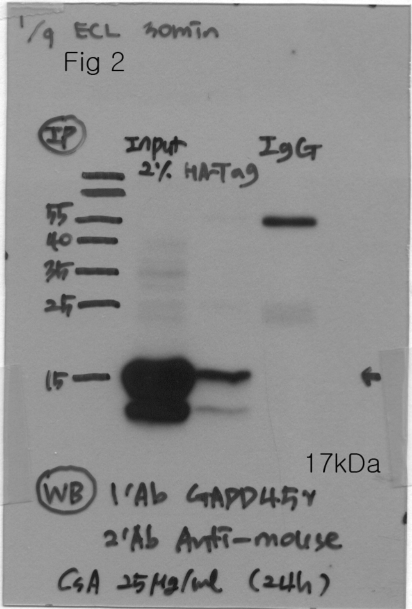
1/9 ECL 30min
Fig 2
IP
Input 2%
HA-Tag
IgG
55
40
35
25
15
17kDa
WB
1'Ab GAPDH
2'Ab Anti-mouse
CsA 25μg/ml (24h)

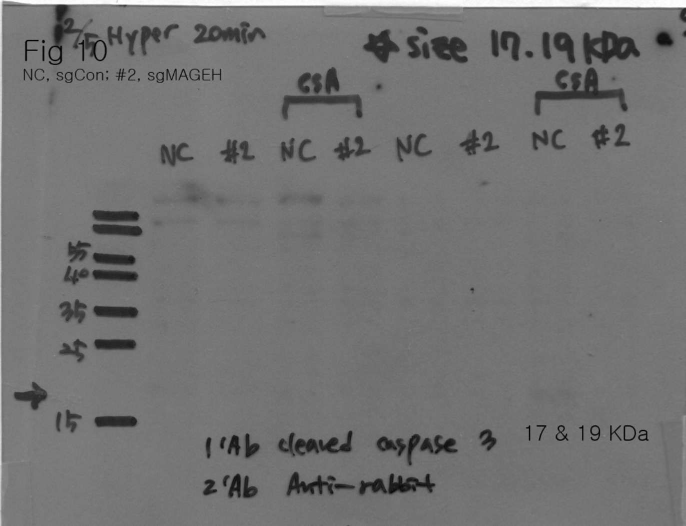
Fig 10
NC, sgCon; #2, sgMAGEH
Hyper 20min
size 17.19 KDa
CSA
CSA
NC #2 NC #2 NC #2 NC #2
55
40
35
25
15
1'Ab cleaved caspase 3
2'Ab Anti-rabbit
17 & 19 KDa

Fig 10

NC,sgCon; #2,sgMAGEH

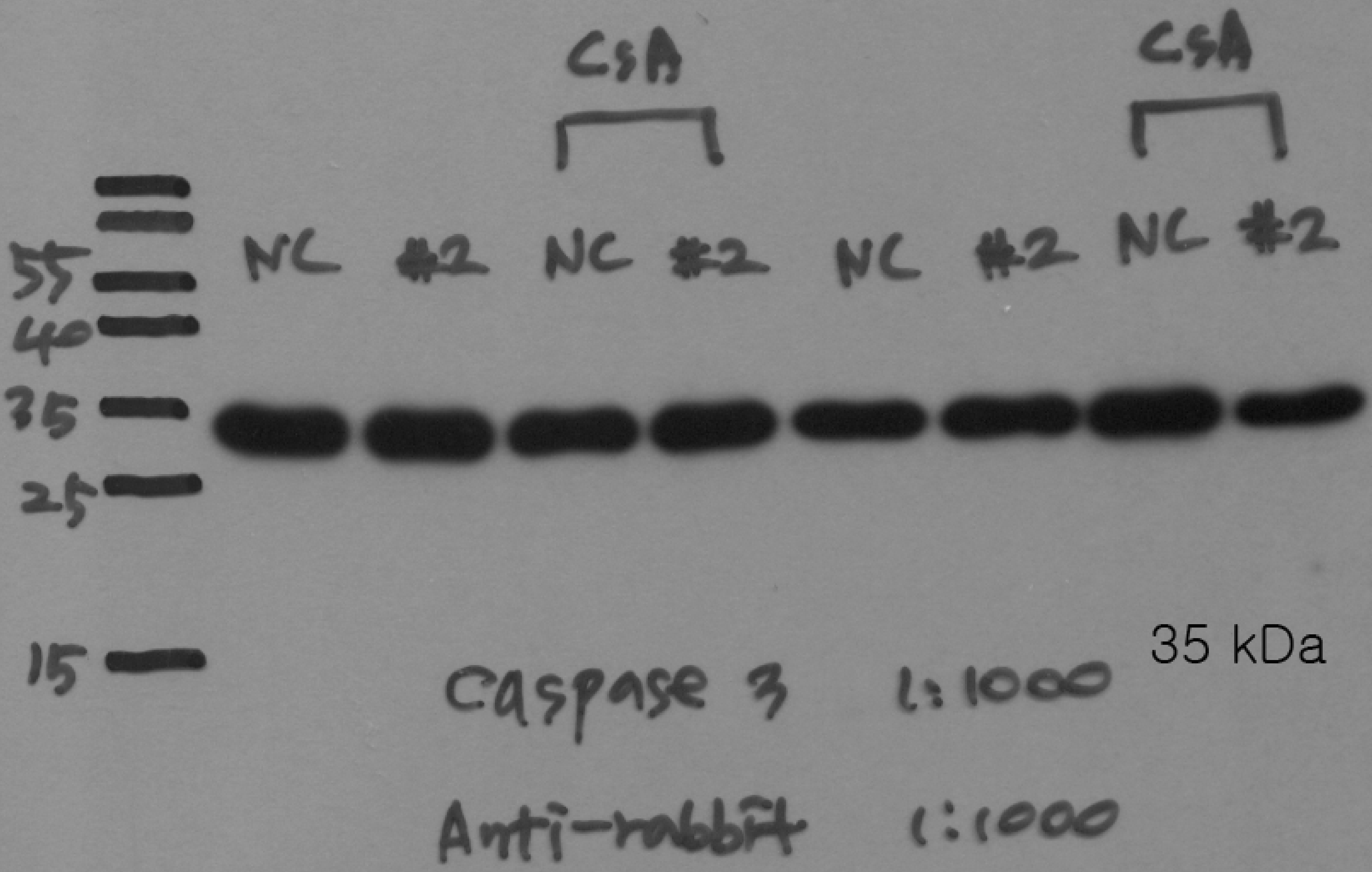

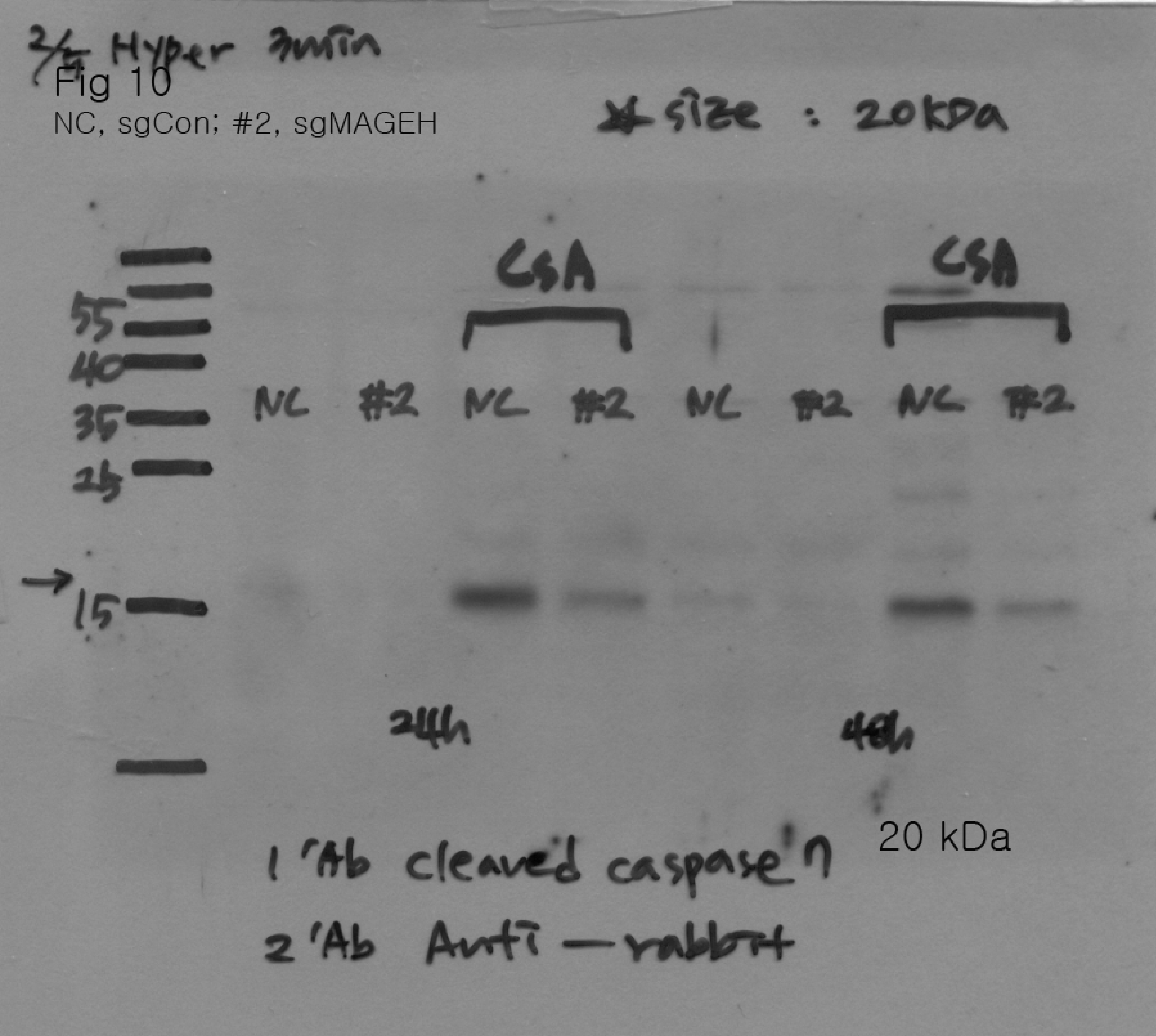

Fig 10

NC, sgCon; #2, sgMAGEH

Fig 10

NC, sgCon; #2, sgMAGEH

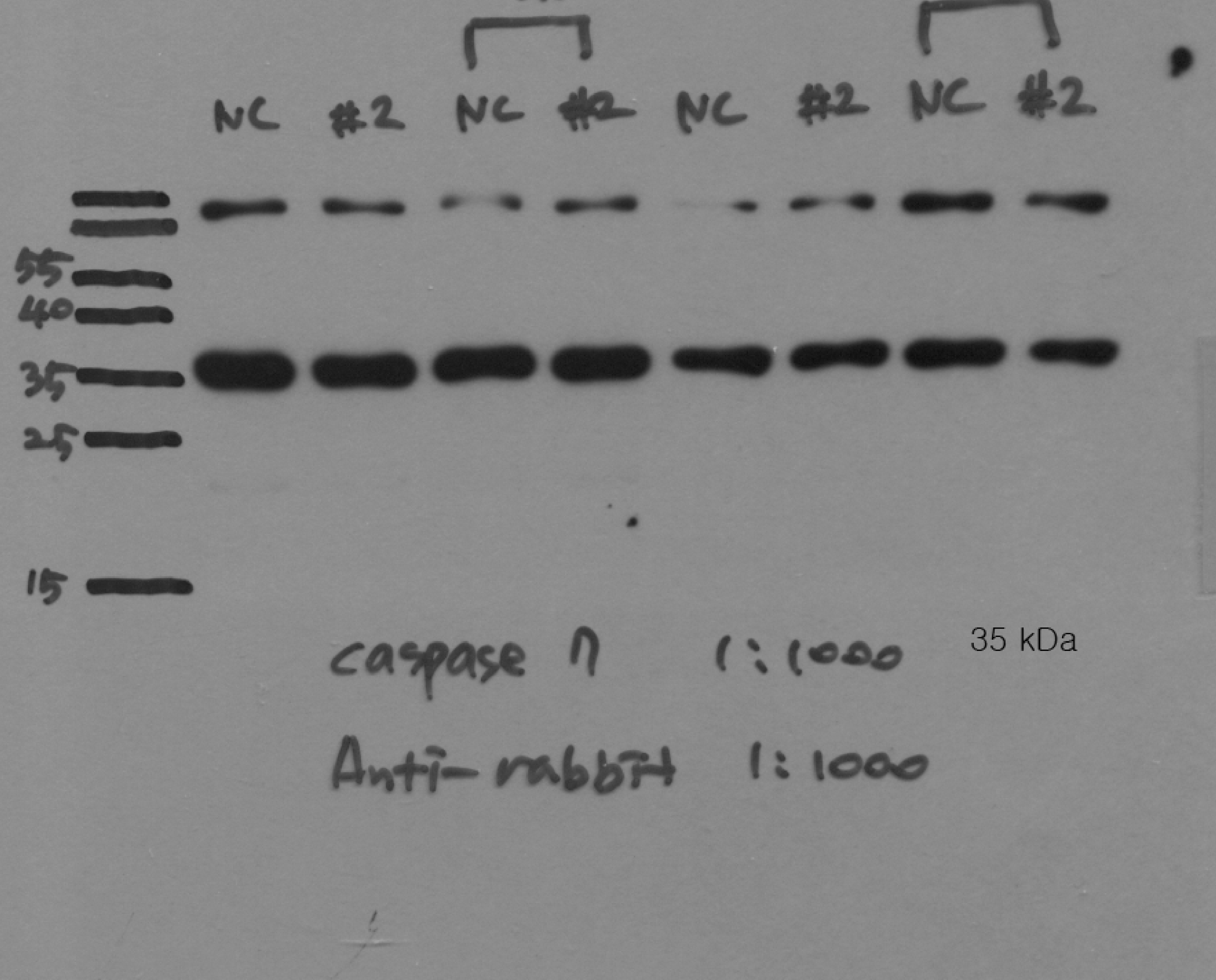

35 kDa

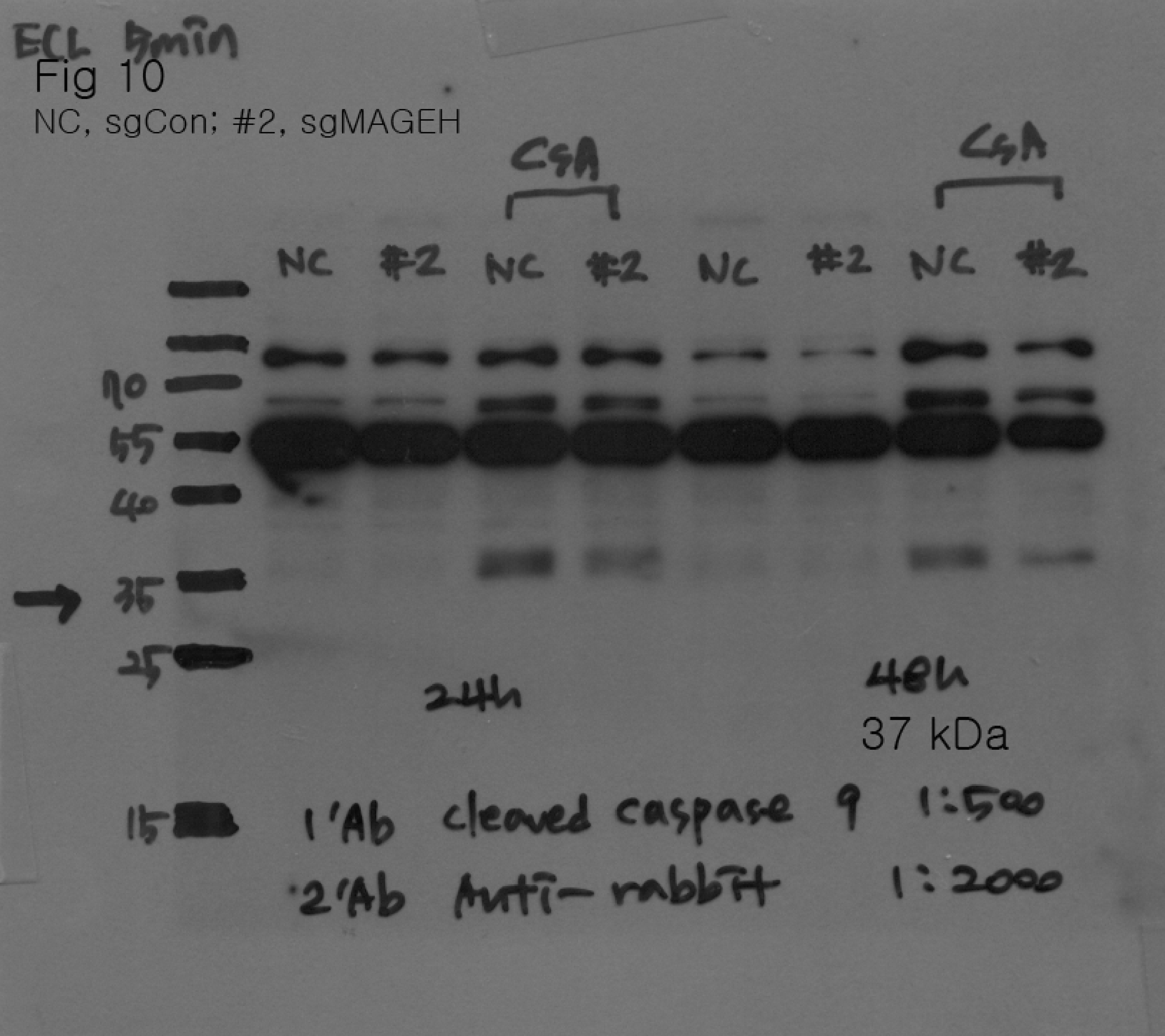

Fig 10
NC, sgCon; #2, sgMAGEH

Fig 10

NC, sgCon; #2, sgMAGEH

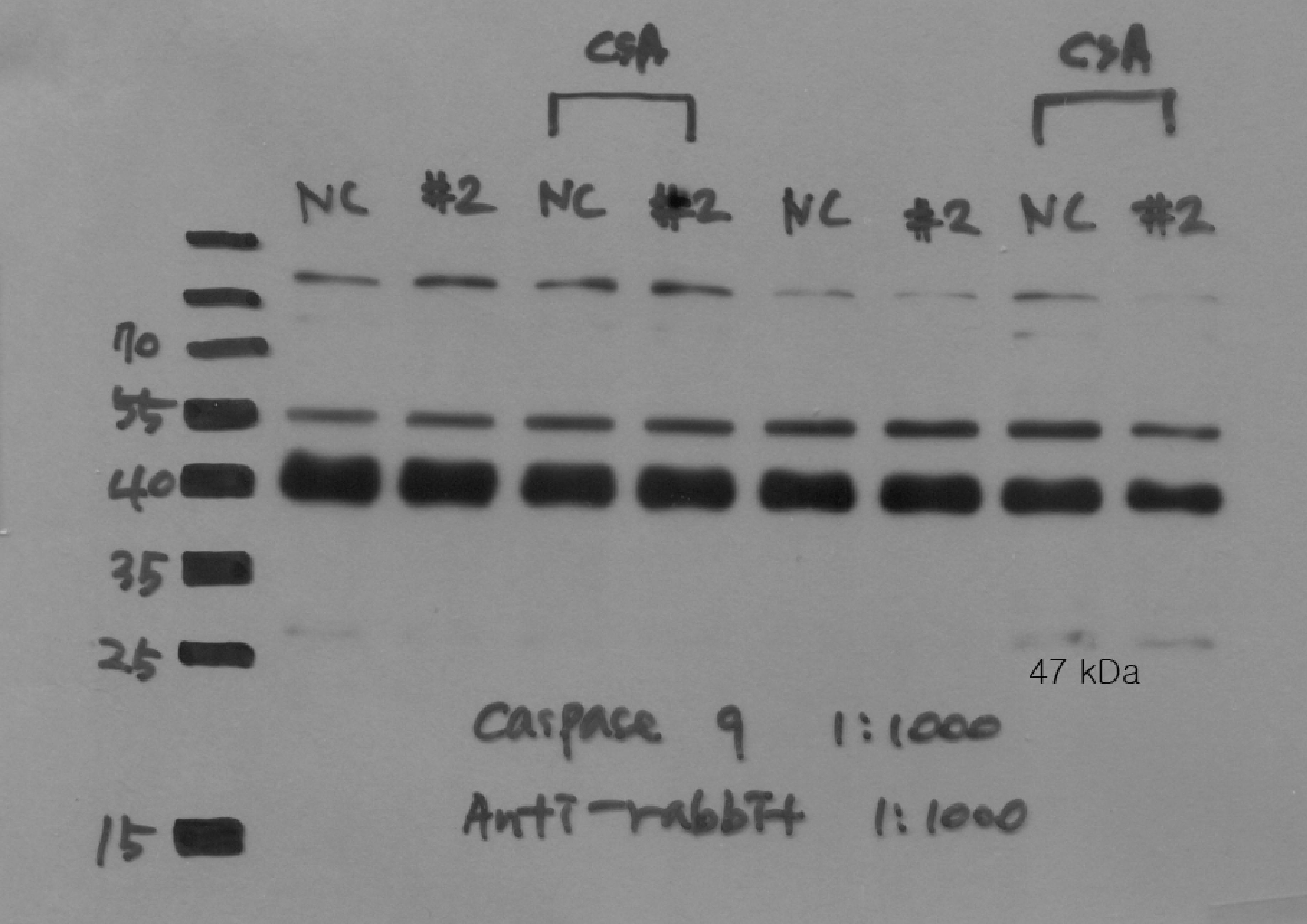

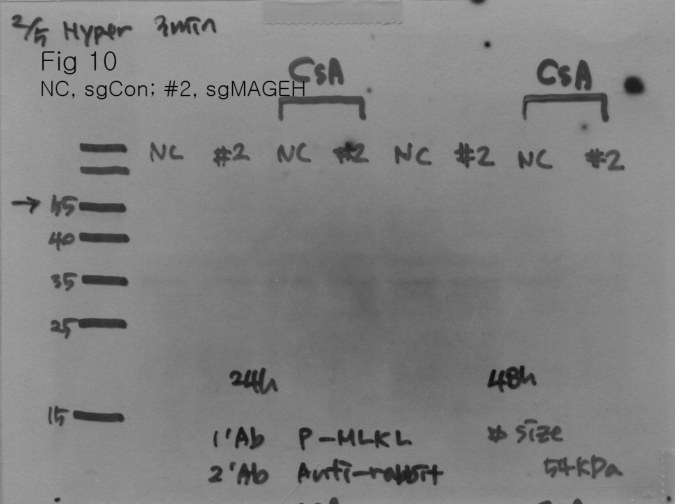

Fig 10

NC, sgCon; #2, sgMAGEH

Fig 10

NC, sgCon; #2, sgMAGEH

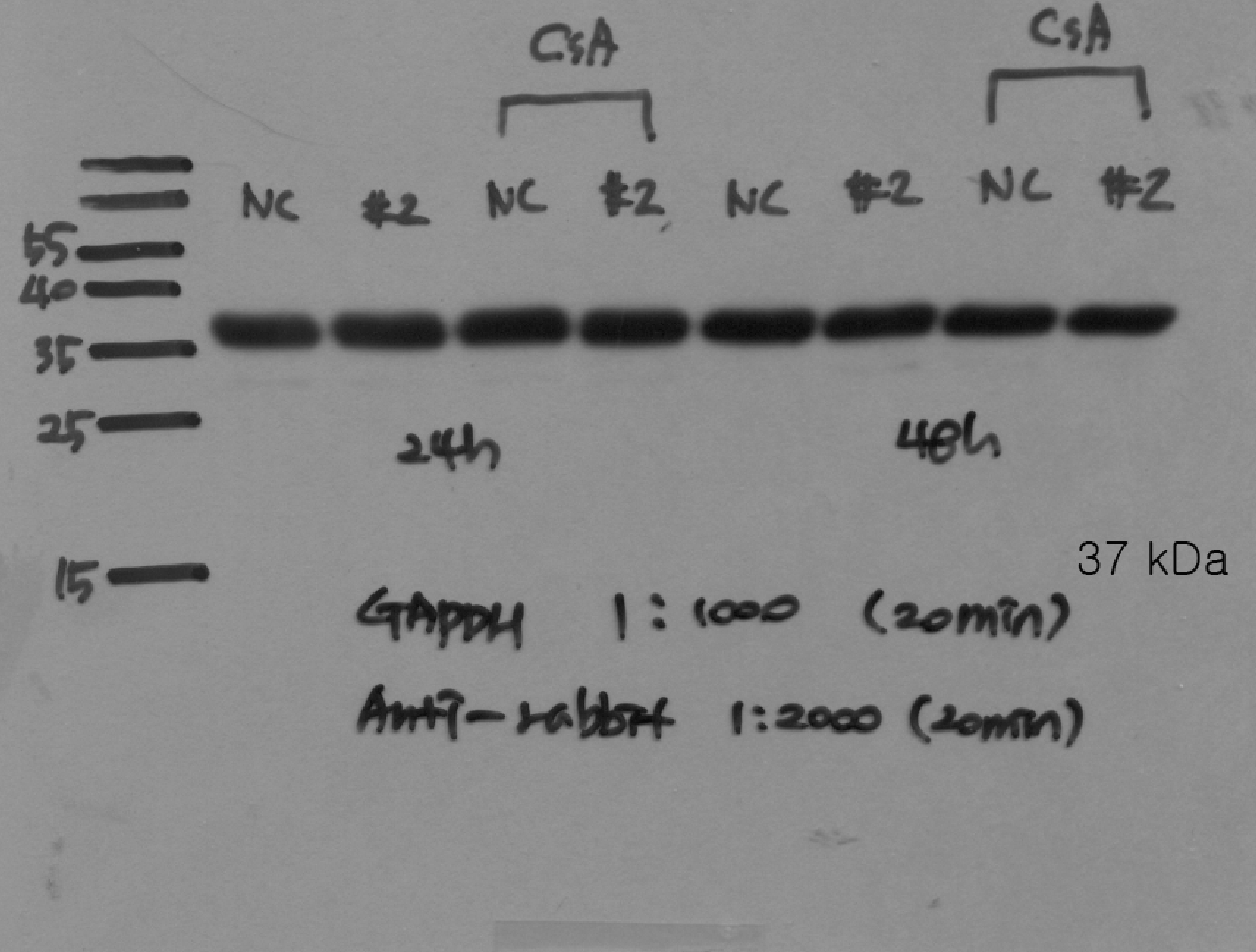

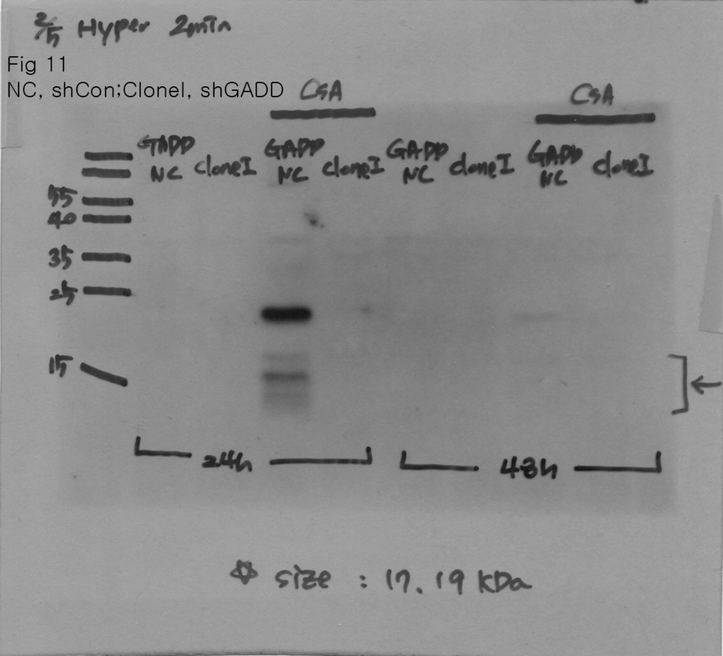

Fig 11
NC, shCon;CloneI, shGADD

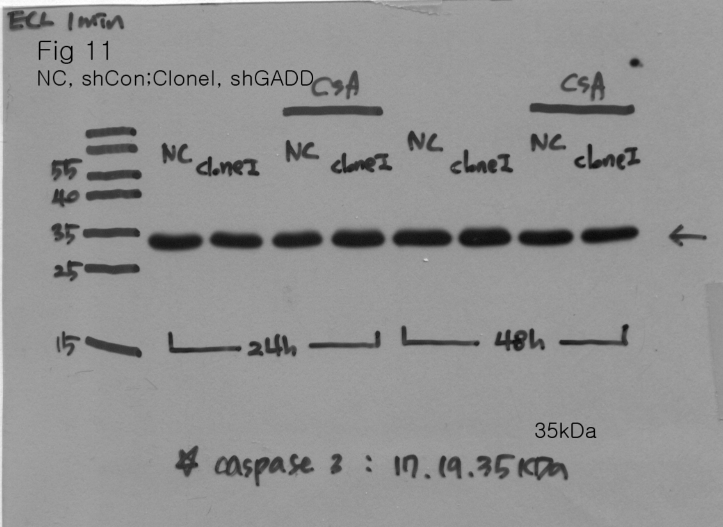
ECL 1min
Fig 11
NC, shCon;CloneI, shGADD
CsA
CsA
55
40
35
25
15
NC
cloneI
NC
cloneI
NC
cloneI
NC
cloneI
24h
48h
35kDa
caspase 3 : 17.19.35KDa

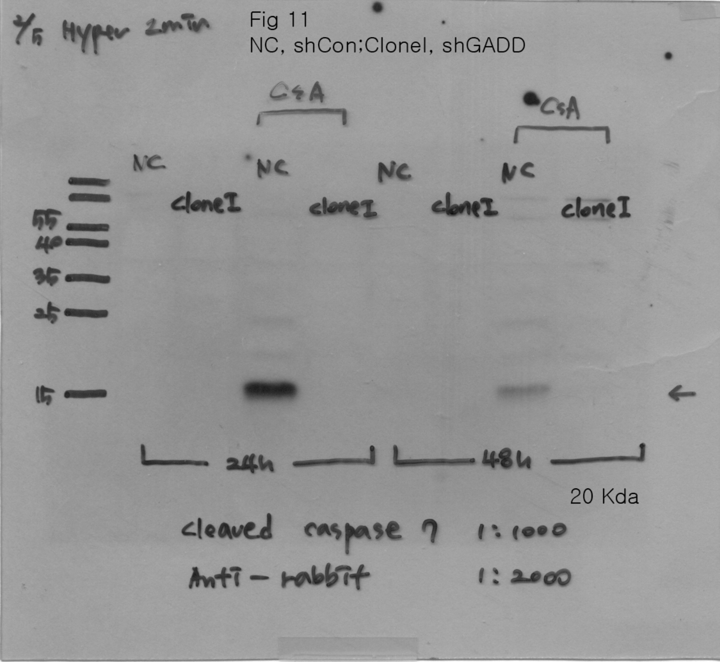

Fig 11
NC, shCon;CloneI, shGADD
3/5 Hyper 2min
CsA
CsA
NC
cloneI
NC
cloneI
NC
cloneI
NC
cloneI
55
40
35
25
15
24h
48h
20 Kda
cleaved caspase 7 1:1000
Anti-rabbit 1:2000

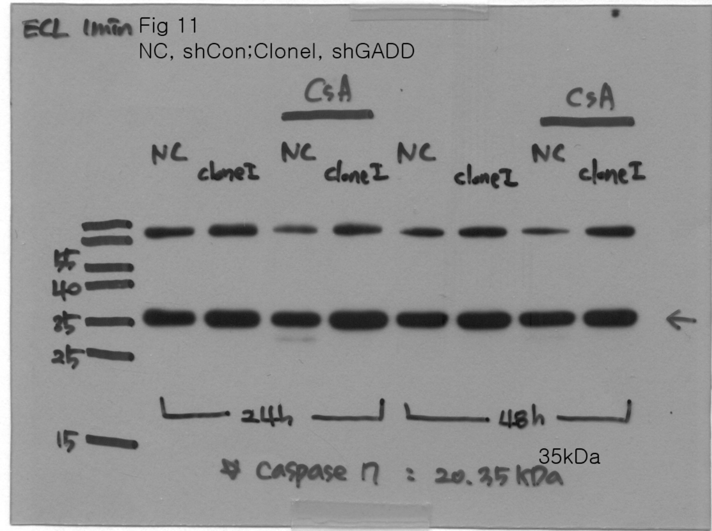

Fig 11
NC, shCon;CloneI, shGADD

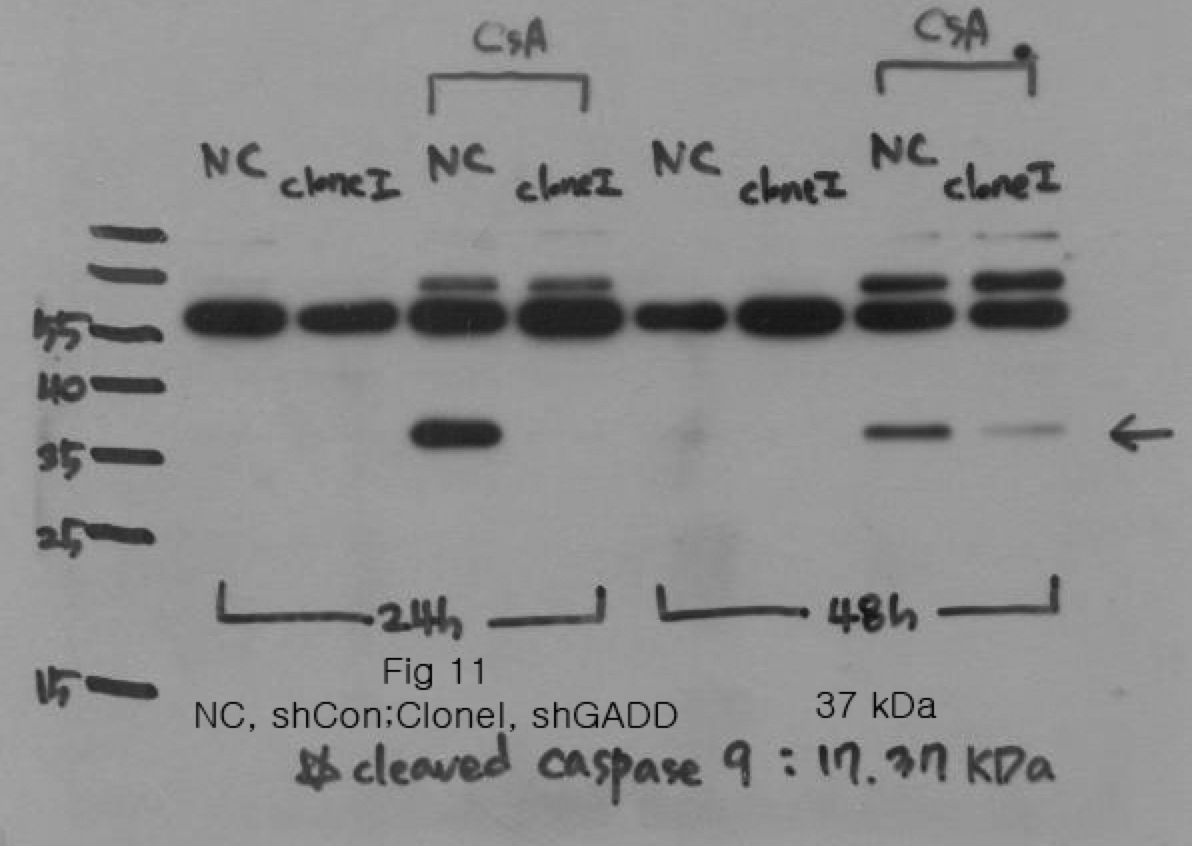

Fig 11
NC, shCon:CloneI, shGADD
37 kDa

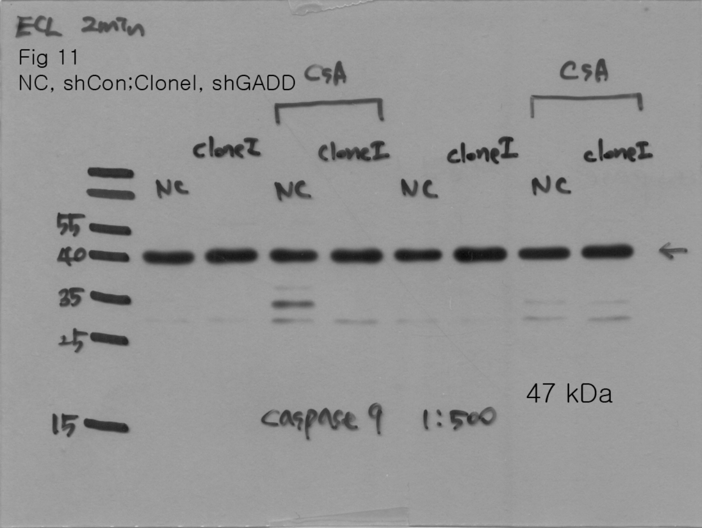
ECL 2min
Fig 11
NC, shCon;CloneI, shGADD
CsA
CsA
cloneI
cloneI
cloneI
cloneI
NC
NC
NC
NC
55
40
35
25
15
47 kDa
caspase 9 1:500

Fig 11

NC, shCon;CloneI, shGADD

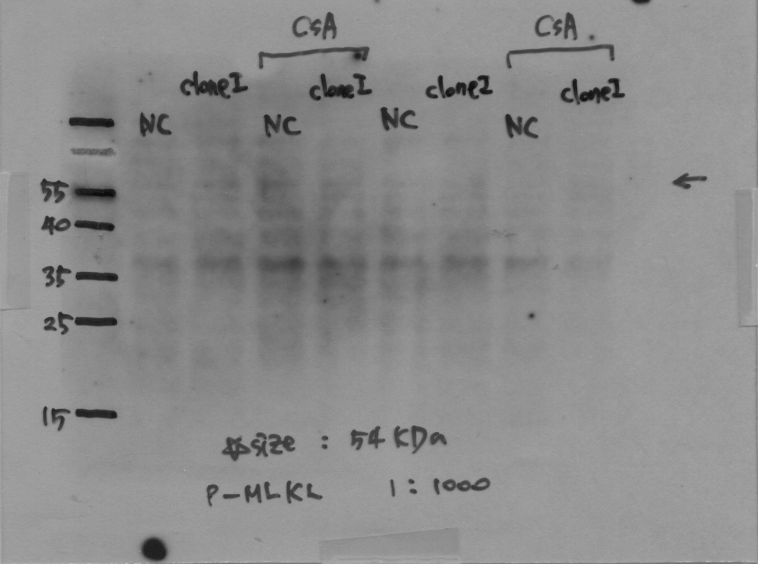

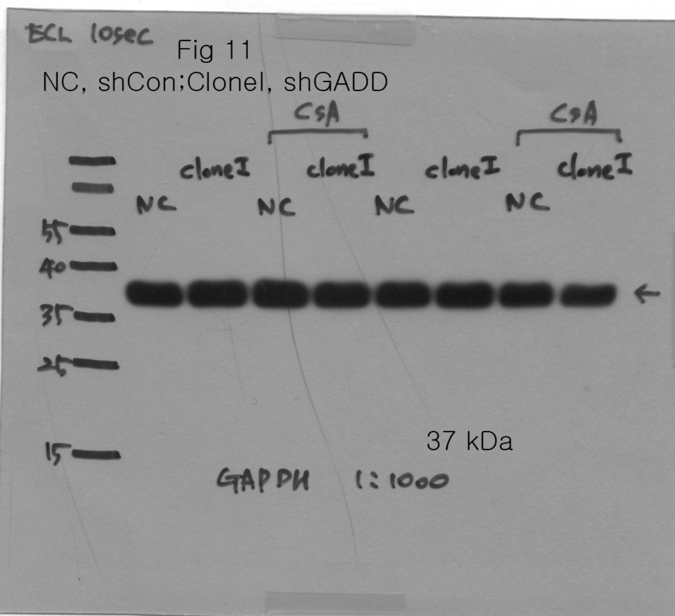
ECL 10sec
Fig 11
NC, shCon;CloneI, shGADD
CsA
CsA
cloneI
cloneI
cloneI
cloneI
NC
NC
NC
NC
55
40
35
25
15
37 kDa
GAPDH 1:1000

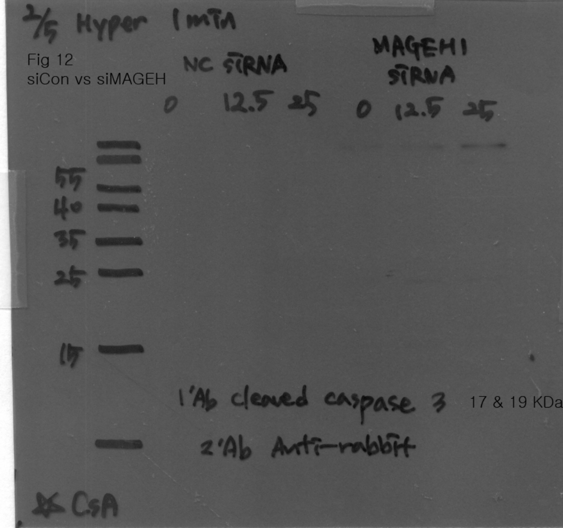
2/5 Hyper 1min
Fig 12
siCon vs siMAGEH
NC siRNA
MAGEH1 siRNA
0 12.5 25
0 12.5 25
55
40
35
25
15
1'Ab cleaved caspase 3
17 & 19 KDa
2'Ab Anti-rabbit
CsA

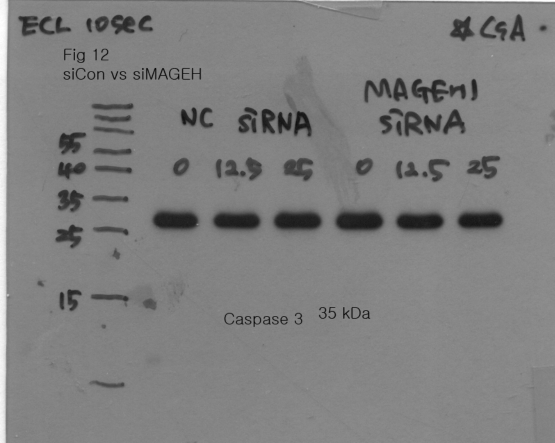

Fig 12
siCon vs siMAGEH

Caspase 3 35 kDa

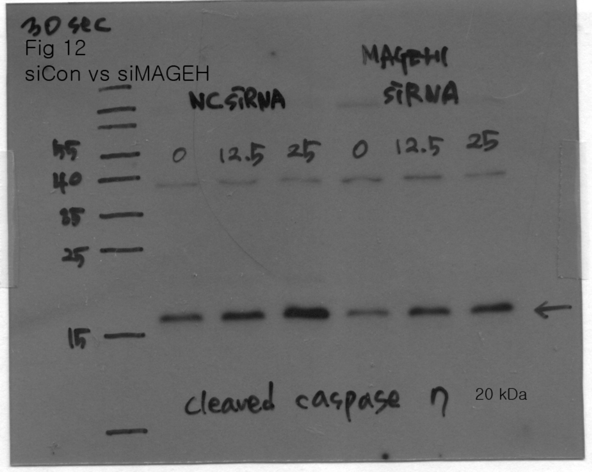
30 sec
Fig 12
siCon vs siMAGEH
NC siRNA
MAGEH1 siRNA
0 12.5 25 0 12.5 25
55
40
35
25
15
cleaved caspase 7
20 kDa

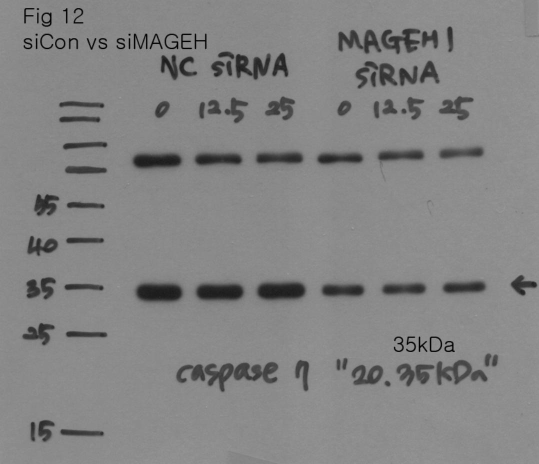

Fig 12
siCon vs siMAGEH

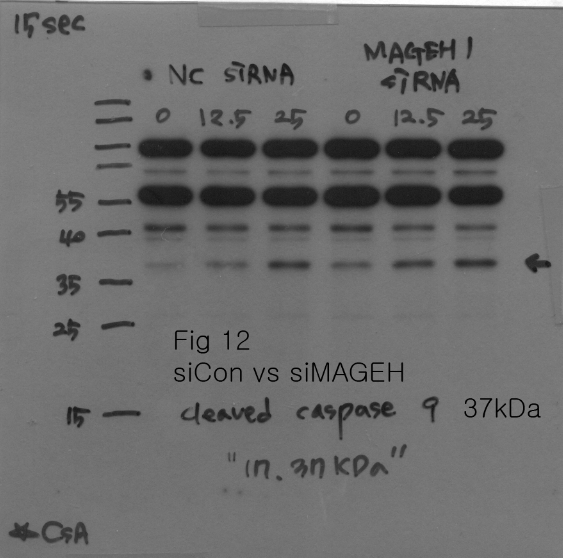
15 sec
NC siRNA
MAGEH1 siRNA
0 12.5 25 0 12.5 25
55
40
35
25
15
Fig 12
siCon vs siMAGEH
cleaved caspase 9 37kDa
"17, 37 kDa"
CsA

Fig 12
siCon vs siMAGEH

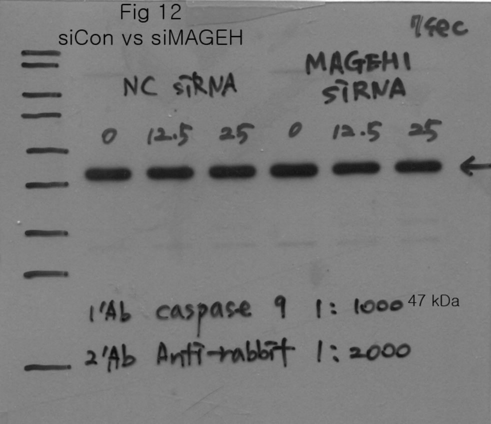

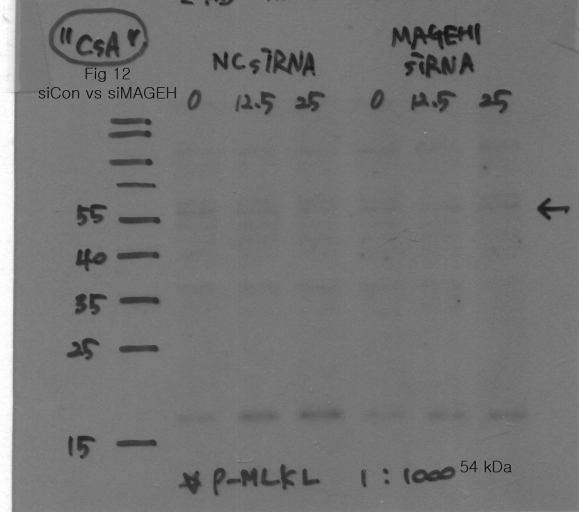

Fig 12
siCon vs siMAGEH
54 kDa

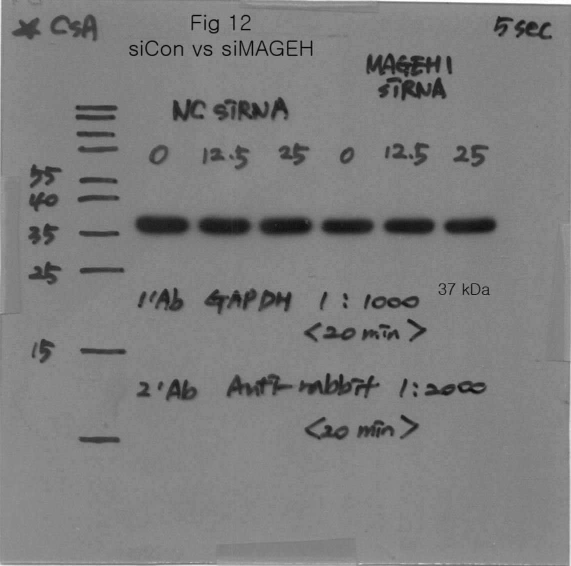
* CsA
Fig 12
siCon vs siMAGEH
5 sec
MAGEH1 siRNA
NC siRNA
0
12.5
25
0
12.5
25
55
40
35
25
15
37 kDa
1'Ab GAPDH 1 : 1000
<20 min>
2'Ab Anti-rabbit 1:2000
<20 min>

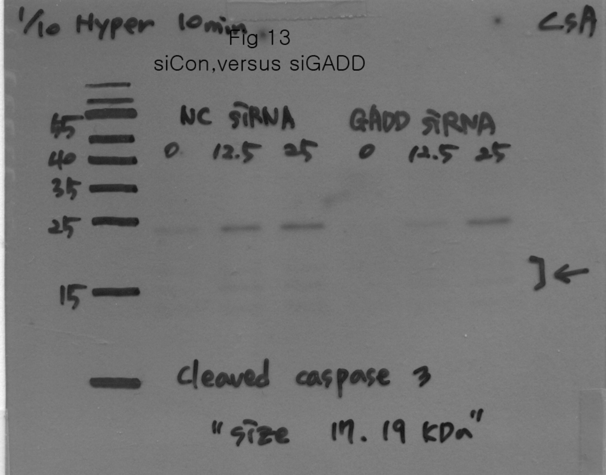

Fig 13
siCon,versus siGADD

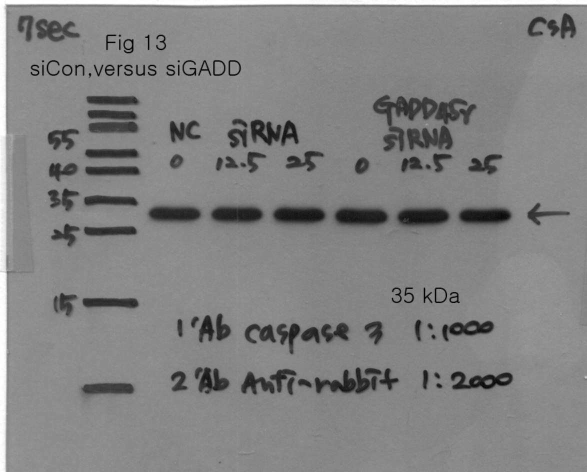

Fig 13
siCon,versus siGADD

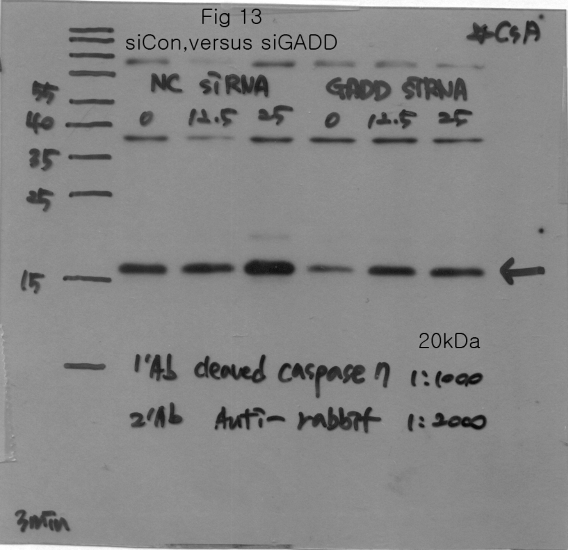
Fig 13
siCon,versus siGADD
#CsA
NC siRNA
GADD siRNA
55
40
35
25
15
0
12.5
25
0
12.5
25
20kDa
1'Ab cleaved caspase 7 1:1000
2'Ab Anti-rabbit 1:2000
3min

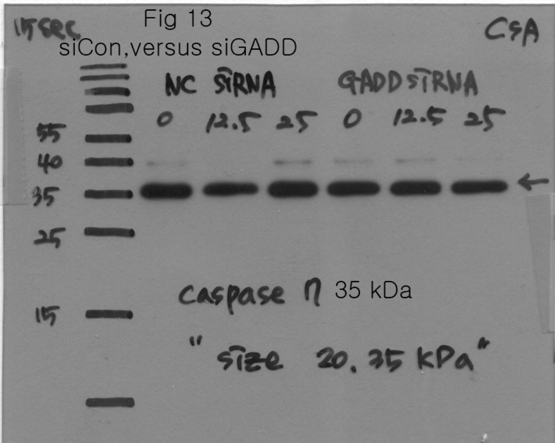
Fig 13
siCon,versus siGADD
NC siRNA
GADD siRNA
0 12.5 25 0 12.5 25
55
40
35
25
15
Caspase
35 kDa
"size 20, 35 kPa"

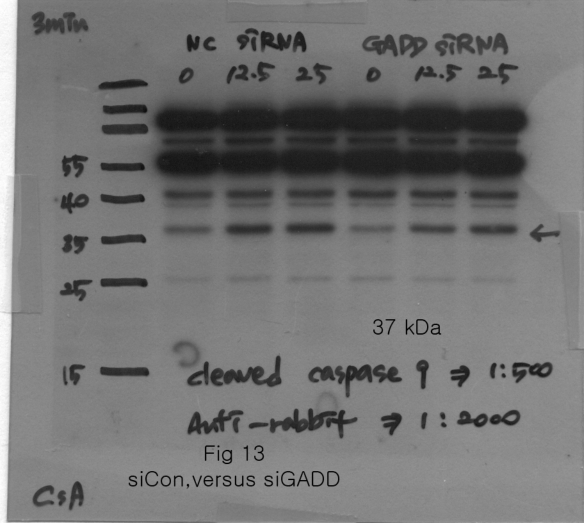

Fig 13
siCon,versus siGADD

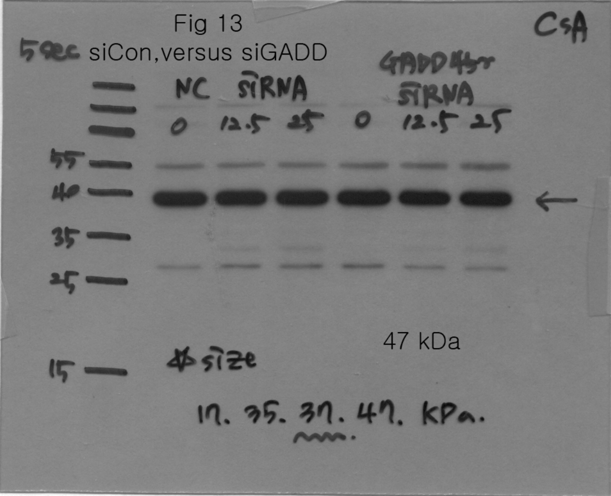
Fig 13
5sec
siCon,versus siGADD
CsA
NC siRNA
siRNA
0
12.5
25
0
12.5
25
55
40
35
25
15
47 kDa
size
17. 35. 37. 47.

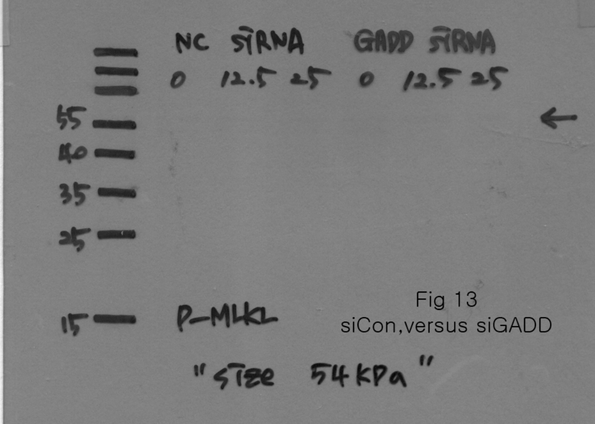

Fig 13
siCon,versus siGADD

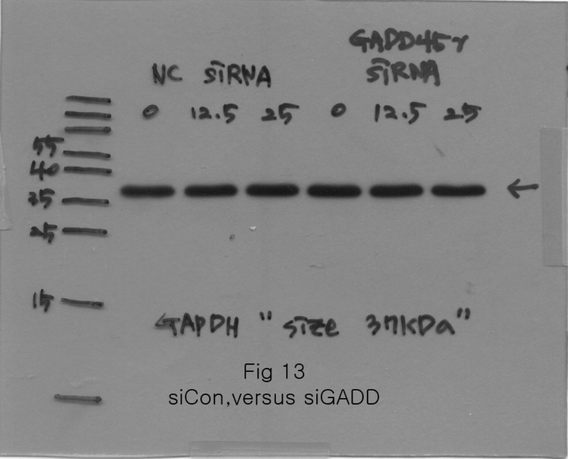

Fig 13
siCon,versus siGADD
